# Synthetic bacterial vesicles combined with tumour extracellular vesicles as cancer immunotherapy

**DOI:** 10.1002/jev2.12120

**Published:** 2021-07-03

**Authors:** Kyong‐Su Park, Kristina Svennerholm, Rossella Crescitelli, Cecilia Lässer, Inta Gribonika, Jan Lötvall

**Affiliations:** ^1^ Krefting Research Centre Institute of Medicine University of Gothenburg Gothenburg Sweden; ^2^ Department of Anesthesiology and Intensive Care Medicine Institute of Clinical Science Sahlgrenska Academy University of Gothenburg Gothenburg Sweden; ^3^ Department of Microbiology and Immunology Institute of Biomedicine University of Gothenburg Gothenburg Sweden

**Keywords:** cancer immunotherapy, synthetic bacterial vesicles, tumour tissue extracellular vesicles

## Abstract

Bacterial outer membrane vesicles (OMV) have gained attention as a promising new cancer vaccine platform for efficiently provoking immune responses. However, OMV induce severe toxicity by activating the innate immune system. In this study, we applied a simple isolation approach to produce artificial OMV that we have named Synthetic Bacterial Vesicles (SyBV) that do not induce a severe toxic response. We also explored the potential of SyBV as an immunotherapy combined with tumour extracellular vesicles to induce anti‐tumour immunity. Bacterial SyBV were produced with high yield by a protocol including lysozyme and high pH treatment, resulting in pure vesicles with very few cytosolic components and no RNA or DNA. These SyBV did not cause systemic pro‐inflammatory cytokine responses in mice compared to naturally released OMV. However, SyBV and OMV were similarly effective in activation of mouse bone marrow‐derived dendritic cells. Co‐immunization with SyBV and melanoma extracellular vesicles elicited tumour regression in melanoma‐bearing mice through Th‐1 type T cell immunity and balanced antibody production. Also, the immunotherapeutic effect of SyBV was synergistically enhanced by anti‐PD‐1 inhibitor. Moreover, SyBV displayed significantly greater adjuvant activity than other classical adjuvants. Taken together, these results demonstrate a safe and efficient strategy for eliciting specific anti‐tumour responses using immunotherapeutic bacterial SyBV.

## 1 INTRODUCTION

Cancer is a persistently increasing concern facing aging populations, and in particular the growing incidence of melanoma leads to nearly 280,000 new cases and over 60,000 deaths worldwide (Ferlay et al., 2019). Immunotherapy breakthroughs over the last decade have shown great promise (Yang, 2015), for example, the regression of cancer has been observed using immunotherapeutic approaches with checkpoint blockade inhibitors, including αPD‐1 and αCTLA‐4 antibodies, and these have been approved for clinical use (Pardoll, 2012). However, these drugs have limited response rates in cancer patients, thus, requiring new complementary approaches.

Tumours express many mutated molecules that can potentially be seen by the immune system as ‘non‐self’, and researchers have explored different approaches to developing immunotherapies targeting these non‐self‐tumour antigens. Some are developing neoantigen‐specific peptides or proteins, but this can be challenging because individual patients can express very different neoantigens. Therefore, using patient tumour material such as tumour cell lysates has been explored, but clinical trials have had only limited success. We hypothesize that tumour extracellular vesicles express large quantities of tumour neoantigens that can be used for the development of individualized immunotherapy approaches. Extracellular vesicles are nanometer‐scale packages of proteins and genetic materials such as mRNA, miRNA and DNA enclosed within lipid bilayers (Gho & Lee, 2017; Lässer et al., 2018; Syn et al., 2017), and our group has recently developed a straightforward technique to isolate extracellular vesicles directly from human melanoma tissues (Jang et al., 2019).

Naturally produced outer membrane vesicles (OMV) from gram‐negative bacteria are spherical and bilayered proteolipids with an average diameter of 20–200 nm (Kim et al., 2015; Schwechheimer & Kuehn, 2015), and they carry large quantities of lipopolysaccharide (LPS), which means that they are very strong inducers of inflammation. OMV also carry other immunostimulatory proteins and can induce an immune response against the bacteria that produced the OMV or when given to tumour‐bearing mice (Acevedo et al., 2014; Toyofuku et al., 2019). Indeed, even though OMV can reduce tumour growth, they are known to provoke severe innate immunity responses *in vivo* such as sepsis, cardiomyopathy and pulmonary diseases via specific subsets of vesicular proteins, possibly even leading to death (Park et al., 2010; Park et al., 2013; Svennerholm et al., 2017).

In this study, we hypothesized that detoxified OMV‐like vesicles could be produced by specific biochemical processes in *Escherichia coli* membranes and that these synthetic bacterial vesicles (SyBV) could be used in combination with tumour tissue‐derived extracellular vesicles (tEV) to induce an active immune response against melanoma and colon cancer. To test this hypothesis, we developed a protocol to co‐inject SyBV and tEV as an immunotherapy in melanoma and colon cancer‐bearing mice with the hypothesis that the combination, but not either component by itself, would induce an immune response that would attenuate tumour growth.

## 2 MATERIALS AND METHODS

### 2.1 Animals

Wild‐type mice of the C57BL/6 genetic background (6 weeks old) were obtained from Charles River. The mice were raised at Experimental Biomedicine (EBM) at the University of Gothenburg, Sweden. The study was approved by the local Animal Ethics Committee in Gothenburg, Sweden (Dnr 5.8.18‐03598/2019), and was carried out according to institutional animal use and care guidelines.

### 2.2 Cell culture

B16F10, a murine melanoma cell line, was purchased from ATCC (Manassas, VA, USA) and maintained in Iscove's Modified Dulbecco's Medium (Thermo Fisher Scientific, Waltham, MA, USA) containing 10% fetal bovine serum (FBS), 1 mM sodium pyruvate, 100 U/ml penicillin, and 100 μg/ml streptomycin. CT26, a murine colon carcinoma cell line, was purchased from ATCC and cultured in in RPMI 1640 medium (HyClone, Logan, UT, USA) containing 10% FBS, 2 mM L‐glutamine, 100 U/ml penicillin, and 100 μg/ml streptomycin. RAW 264.7 cells were grown in Dulbecco's modified Eagle's medium (HyClone) supplemented with 10% FBS, 100 U/ml penicillin, and 100 μg/ml streptomycin. Mouse bone marrow‐derived dendritic cells (BMDCs) were isolated as previously described (Lutz et al., 1999). Briefly, bone marrow cells were harvested from the femur and the tibia of mice. The cells were differentiated into DCs by incubating in RPMI 1640 medium supplemented with 10% FBS, 50 μM β‐mercaptoethanol, 20 ng/ml GM‐CSF, 100 U/ml penicillin, and 100 μg/ml streptomycin for 1 week. All cells were cultured at 37°C in an atmosphere of 5% CO_2_.

### 2.3 Preparation of SyBV

*E. coli* outer membranes were first purified from culture supernatants as described previously with some modifications (Lee et al., 2007). The bacterial cultures were pelleted, resuspended in 20 mM Tris‐HCl (pH 8.0) with 20% sucrose, and treated with lysozyme (600 μg per g cells) and 0.1 M EDTA (0.2 ml per g cells). The resulting spheroplasts were pelleted and then sonicated in 10 mM Tris‐HCl (pH 8.0) at 4°C by using Q55 Sonicator (20 kHz, QSonica, Newtown, CT). The amplitude of the sonicator was set to 40 and the sonication was performed for 2 min with a 3 mm in diameter ultrasound probe. The unbroken cells were removed by centrifuging at 8000 × *g* for 5 min, and then whole membranes were pelleted from the supernatants at 40,000 × *g* for 1 h at 4°C. The membranes were incubated in 0.5% Sarkosyl (Sigma Aldrich, St. Louis, MO, USA) for 20 min, and the outer membranes were pelleted at 40,000 × *g* for 1 h at 4°C. Next, the pellets were incubated with high pH solution (200 mM Na_2_CO_3_, pH 11) for 1 h at 25°C. The solutions were applied to 4 ml of 50% iodixanol (Axis‐Shield PoC AS, Oslo, Norway) followed by addition of 4 ml of 30% iodixanol and 2 ml of 10% iodixanol in an ultracentrifuge tube. The layers formed between 10% and 30% iodixanol after ultracentrifugation at 100,000 × *g* for 2 h at 4°C were collected. Finally, the samples were sonicated for 30 min with an ultrasonic bath of 44 kHz (Grant, Cambridge, UK) and considered as SyBV.

### 2.4 Preparation of natural OMV

OMV derived from *E. coli* were isolated using the protocol described previously with modification (Lee et al., 2007). Bacterial cultures were pelleted at 6000 × *g* at 4°C for 20 min twice, and then the supernatant was filtered through a 0.45‐μm vacuum filter (Corning, Corning, NY, USA). The filtered solution was concentrated using a Vivaflow 200 module (Sartorius, Goettingen, Germany) with a 100 kDa cut‐off membrane. The remaining solution was subjected to ultracentrifugation at 150,000 × *g* at 4°C for 3 h, and the pellets were resuspended with phosphate‐buffered saline (PBS).

### 2.5 Transmission electron microscopy (TEM)

SyBV were investigated by negative stain electron microscopy. SyBV were placed for 5 min on glow‐discharged formvar carbon‐coated 200‐mesh copper grids (Electron Microscopy Sciences, Hatfield, PA, USA). The SyBV were then washed with water, followed by fixing with 2.5% glutaraldehyde dissolved in PBS. After washing again with water, the samples were stained with 2% uranyl acetate for 1.5 min. Negative‐stained vesicles were observed with an LEO 912AB Omega electron microscope (Carl Zeiss SMT, Oberkochen, Germany) at 120 kV with a Veleta CCD camera (Olympus‐SiS, Stuttgart, Germany).

### 2.6 Nanoparticle tracking analysis

SyBV (10 μg/ml) were dissolved in PBS, and the particle concentration of SyBV was measured with a ZetaView analyser (Particle Metrix GmbH, Meerbuch, Germany). Measurements were performed in triplicate, and each individual data point was acquired from two stationary layers with five measurements in each layer. The sensitivity of the camera was set to 70 for all measurements. Data were analysed using ZetaView analysis software version 8.2.30.1.

### 2.7 RNA and DNA analysis

RNA from SyBV or OMV was isolated using the miRCURY RNA isolation kit for biofluids (Exiqon, Vedbaek, Denmark) according to the manufacturer's protocol. Also, DNA was isolated using the Qiamp DNA Blood Mini kit (Qiagen, Hilden, Germany) according to the manufacturer's protocol. One microliter of isolated RNA and DNA was analysed for its quality, yield, and nucleotide length by capillary electrophoresis using an Agilent RNA 6000 Nanochip and Agilent High sensitivity DNA chip, respectively, on an Agilent 2100 Bioanalyzer (Agilent Technologies GmbH, Berlin, Germany).

### 2.8 LC‐MS/MS analysis

Two biological replicates of SyBV and OMV (30 μg) were digested with trypsin using the filter‐aided sample preparation (FASP) method and C18 spin columns according to the manufacturer's instructions (Wiśniewski et al., 2009). All fractions were dried on a Speedvac and reconstituted in 3% acetonitrile and 0.2% formic acid and analysed on an Orbitrap Fusion Tribrid mass spectrometer interfaced with an Easy‐nLC 1200 (Thermo Fisher Scientific, Waltham, MA, USA). Peptides were trapped on an Acclaim Pepmap 100 C18 trap column (100 μm × 2 cm, particle size 5 μm; Thermo Fischer Scientific) and separated on an in‐house packed C18 analytical column (75 μm × 30 cm, particle size 3 μm) using a gradient from 5% to 33% B over 160 min and from 33% to 100% B over 5 min. Solvent A was 0.2% formic acid, and solvent B was 80% acetonitrile and 0.2% formic acid. Precursor ion mass spectra were recorded at 120,000 resolution, and the most intense precursor ions were fragmented using HCD at a collision energy setting of 30. The MS/MS spectra were recorded at 30,000 resolution with a maximum injection time of 125 ms and an isolation window of 1.0 Da. Charge states 2–7 were selected for fragmentation, and dynamic exclusion was set to 45 s with 10 ppm tolerance.

### 2.9 Database search

Data analysis was performed with Proteome Discoverer version 1.4 (Thermo Fisher Scientific). The database search was executed against the Swissprot *E. coli* database. Mascot 2.5.1 (Matrix Science, London, UK) was utilized as a search engine with precursor mass tolerance of 5 ppm and fragment mass tolerance of 0.5 Da, and one missed cleavage was accepted, mono‐oxidation on methionine was set as a variable modification, and methylthiolation on cysteine was set as a fixed modification. Target Decoy PSM validator was used for the validation of identification results with the strict target false discovery rate of 1%. Gene ontology analysis was obtained using the Funrich analysis tool, and principal component analysis and hierarchical cluster analysis were accomplished with ClustVis software. The mass spectrometry data has been deposited to the ProteomeXchange Consortium via the PRIDE partner repository with the dataset identifier PXD025421.

### 2.10 SDS‐PAGE

Bacterial whole‐cell lysates, outer membrane proteins (OMPs) and periplasmic proteins were made from *E. coli* culture according to a previously reported method (Lee et al., 2007). These proteins together with OMV and SyBV were separated by 10% SDS‐PAGE and transferred to a polyvinylidene difluoride membrane. The blocked membrane was then incubated with anti‐OmpA antibody (MyBioSource, San Diego, CA, USA). After incubation with horseradish peroxidase‐conjugated secondary antibody, the immunoreactive bands were visualized with a chemiluminescent substrate.

### 2.11 Immune toxicity test

*In vitro* immune toxicity of SyBV was tested in cytokine release assays from RAW 264.7 cells. The cells were seeded into 24‐well plates and then various concentrations of SyBV or OMV were treated for 24 h to check pro‐inflammatory cytokines. The levels of tumour necrosis factor (TNF)‐α and interleukin (IL)‐6 in the supernatants were measured by a DuoSet ELISA Development kit (R&D Systems, Minneapolis, MN, USA). For *in vivo* safety assessment, mice were intraperitoneally injected with SyBV (5 × 10^10^ and 25 × 10^10^ particle numbers in 100 μl PBS) or OMV (5 × 10^9^ particle numbers in 100 μl PBS) and then sacrificed at 6 h following anaesthetization with intraperitoneal injection of xylazine chloride (10 mg/kg; Bayer, Gothenburg, Sweden) and ketamine hydrochloride (100 mg/kg; Pfizer AB, Kent, UK). Rectal temperature was measured with a thermometer (Bioseb, Chaville, France). For leukocyte and platelet counting, blood was acquired by cardiac puncture and kept in EDTA tubes. The numbers of leukocytes and platelets were counted using the EVOS XL Core Imaging System (Life Technologies, Bothell, WA, USA) following incubation with 1% hydrochloride and Rees–Ecker diluting fluid (Thermo Fisher Scientific), respectively. Peritoneal fluid and bronchoalveolar lavage (BAL) fluid were collected from mice and then the supernatants were kept at −80°C for cytokine analysis after centrifugation.

### 2.12 Toll‐like receptor (TLR) screening assay

Recombinant HEK‐293 cell lines overexpressing various murine TLR were provided by InvivoGen (Toulouse, France). The receptors were engaged with a reporter gene which is a secreted alkaline phosphatase. The production of this reporter gene was driven by a NF‐ĸB inducible promoter, and then TLR activation results were given as optical density values after 18 h stimulation of the cells with SyBV or OMV.

### 2.13 Uptake of SyBV

SyBV labelled with DiO (Molecular Probes, Eugene, OR, USA) were incubated with Cellmask Deep Red (Thermo Fisher Scientific)‐labelled BMDCs for 3, 6, or 12 h. The cells were fixed with 4% paraformaldehyde and then permeabilized with 0.2% Triton X‐100. The uptake was analysed by a fluorescence light microscope (Zeiss Axio Observer; Carl Zeiss) following mounting with Prolong Gold antifade reagent (Thermo Fisher Scientific). For the uptake inhibitor treatment, BMDCs pretreated with dynasore (Sigma Aldrich, St. Louis, MO, USA) were incubated with DiO‐labelled SyBV for 6 h. Flow cytometry was analysed using a BD FACSVerse Flow Cytometer running BD FACSuit Software (BD Biosciences, San Jose, CA) and FlowJo Software (Tree Star Inc., Ashland, OR, USA).

### 2.14 Dendritic cell maturation analysis

BMDCs treated with SyBV for 24 h were blocked for non‐specific staining with 2.4G2 (anti‐Fc‐receptor) for 30 min at 4°C in PBS/0.1% BSA and then washed and stained for 30 min at 4°C in PBS/0.1% BSA with the following antibodies: rat anti‐mouse MHCII‐ BV421 obtained from eBioscience; rat anti‐mouse CD11c‐PE‐Cy7, rat anti‐mouse CD83‐PE (Michel‐19), and rat anti‐mouse CD40‐APC‐Cy7 obtained from BD Biosciences; and rat anti‐mouse CD86‐BV510 obtained from BioLegend. To exclude dead cells, 7‐aminoactinomycin D (Sigma Aldrich)‐stained cells were excluded from the analysis. Events were collected and analysed using a Fortessa‐X20 Flow cytometer (BD Biosciences) and FlowJo Software (Tree Star Inc.). For signalling molecule analysis, BMDCs were treated with SyBV plus tEV for 5–30 min. Whole‐cell lysates were separated by 10% SDS‐PAGE and transferred to a polyvinylidene difluoride membrane. The blocked membrane was then incubated with anti‐beta‐actin antibody (Sigma Aldrich) or anti‐phospho‐p65 (serine 365; Cell Signaling Technology). After incubation with horseradish peroxidase‐conjugated secondary antibody, the immunoreactive bands were visualized with a chemiluminescent substrate.

### 2.15 Isolation of tEV

Extracellular vesicles from tumour tissues were isolated as described previously (Jang et al., 2019). Briefly, tumour pieces from humans or mice were gently sliced into small fragments (1–2 mm) and incubated with Collagenase D (Roche, Indianapolis, IN, USA) (2 mg/ml) and DNase I (Roche) (40 U/ml) for 30 min at 37°C to dissolve fibrotic structures. Cells and tissue debris were eliminated by centrifugation at 300 × *g* for 10 min and 2000 × *g* for 20 min at 4°C following filtration through a 70‐μm cell strainer. Supernatants were ultracentrifuged at 16,500 × *g* for 20 min and 118,000 × *g* for 2.5 h at 4°C to collect large and small vesicles, respectively. Only small vesicles were resuspended in PBS, and these were considered tEV. For human tumour samples, tissues from melanoma lymph node metastases were acquired from patients assigned to stage 3 or 4. The human study was approved by the Regional Ethical Review Board at the University of Gothenburg (096–12), and all participants gave written informed consent. For isolation of extracellular vesicles from RAW 264.7, the cell supernatants were sequentially centrifuged at 300 × *g* for 10 min and 2000 × *g* for 20 min at 4°C to remove cell debris. Then, the resulting supernatants were ultracentrifuged at 16,500 × *g* for 20 min and 118,000 × *g* for 2.5 h at 4°C to collect small vesicles as extracellular vesicles. The vesicles were used for the related experiments after freezing.

### 2.16 Antitumour experiment

Mice were subcutaneously inoculated with B16F10 or CT26 cells (5 × 10^5^) and maintained to form a measurable tumour mass (2–3 mm). Samples (tEV, 5 × 10^9^; SyBV, 5 × 10^9^) were subcutaneously injected into the mice five times at 3‐day intervals. The treated tEV were originated from same melanoma tissues. The tumour size was measured every 1 or 2 days using a calliper, and tumour volume was calculated according to the formula (width)^2^ × (length) / 2. For anti‐PD‐1 immunotherapy, anti‐mouse PD‐1 antibody (100 μg; BioXcell, West Lebanon, NH) was intraperitoneally injected into mice 1 day prior to immunization with tEV and SyBV. For the metastasis assay, mice were intravenously injected with B16F10 cells (1 × 10^5^) and then subcutaneously immunized five times at 3‐day intervals. At day 17, mice were sacrificed and the number of colonies metastasized in the lungs were counted.

### 2.17 Tissue histology

Tumour, lung, liver, heart and kidney were excised and fixed in 4% formaldehyde overnight at room temperature. The tissues were dehydrated and embedded in paraffin, and the paraffin blocks were sectioned to 4 μm thickness and stained with haematoxylin and eosin. The images were obtained using the EVOS XL Core Imaging System (Life Technologies, Bothell, WA, USA).

### 2.18 Immune cell subpopulations by flow cytometry

At day 17, tumours and inguinal lymph nodes were removed upon sacrifice after immunization, and single cell suspensions were made. Cells were counted for absolute numbers using a Muse cell counter (Millipore, Bedford, MA, USA) and stained for surface expression for 30 min at 4°C in PBS/0.1% BSA with the following antibodies: rat anti‐mouse F4/80‐eF660 (BM8) and rat anti‐mouse MHCII‐AF700 (M5/114.15.2) obtained from eBioscience; hamster anti‐mouse CD11c‐PeCy7 (HL3), rat anti‐mouse CD11b‐BV510 (M1/70), rat anti‐mouse Ly6g‐BV650 (1A8), rat anti‐mouse CD19‐PECF594 (1D3), hamster anti‐mouse CD3e‐BUV737 (500A2), rat anti‐mouse NKG2A/C/E‐BV421 (20d5), rat anti‐mouse CD45‐APC‐Cy7 (30‐F11), and rat anti‐mouse CD8a‐FITC (53‐6.7) obtained from BD Biosciences; and rat anti‐mouse CD4‐BV785 (RM4‐5) and rat anti‐mouse Ly6c‐BV605 (HK1.4) obtained from BioLegend. To exclude dead cells, 7‐aminoactinomycin D (Sigma Aldrich)‐stained cells were excluded from the analysis. Events were collected and analysed using a Fortessa‐X20 Flow cytometer (BD Biosciences) and FlowJo Software (Tree Star, Inc.).

### 2.19 Measurement of antibody titre against tumour vesicle proteins

Blood samples were taken from mice 3 days after each immunization and assayed for their antibodies specific for tEV proteins. The mouse serum was diluted 1:500 in 1% BSA/PBS and placed in 96‐well plates coated with 200 ng of tEV. After incubation for 2 h, the antibody levels were measured with a peroxidase‐conjugated anti‐mouse IgG and IgG_2a_ antibody (Thermo Fisher Scientific).

### 2.20 Cytokine release by splenocytes

At day 17, CD4^+^ and CD8^+^ T cells were purified from mouse spleens following immunization using a cell isolation kit (Miltenyi Biotec, Bergish Gladbach, Germany) according to the manufacturer's instructions. The cells (5 × 10^5^) were incubated for 72 h with 1 μg/ml of tEV, followed by quantification of cytokines in the supernatants using a DuoSet ELISA Development kit (R&D Systems, Minneapolis, MN, USA).

### 2.21 Cytotoxicity assay

To acquire effector cytotoxic T lymphocytes (CTLs), isolated splenic T cells were cultured with tEV (1 μg/ml) in the presence of IL‐2 (20 U/ml; Sigma Aldrich). At day 5, live T cells were collected and used as effector cells. B16F10 cells as target cells were seeded into 96‐well plates and incubated with CTL effector cells at effector/target cell ratios of 12.5, 25, or 50. The MTT (3‐[4, 5‐dimethylthiazol‐2‐yl]‐2, 5 diphenyl tetrazolium bromide) assay was used to determine CTL activity for 24 h. The activity was calculated as [1 − (OD of effector and target cells − OD of effector cells) / OD of target cells] × 100%.

### 2.22 Comparison to other traditional adjuvants

Mice were intraperitoneally immunized once a week for 3 weeks with human melanoma tEV (5 × 10^9^) with/without Alum (100 μg; Sigma Aldrich), incomplete Freund's adjuvant (IFA) (50 μl; Sigma Aldrich), and CpG DNA (10 μg; InvivoGen, San Diego, CA), or SyBV (5 × 10^9^) as adjuvants. Blood was obtained from mice 3 days after each immunization and assayed for antibody titre against human tEV proteins using anti‐mouse IgG antibody conjugated with peroxidase (Thermo Fisher Scientific).

### 2.23 Statistical analysis

Data analysis was performed using GraphPad Prism 7. Results were expressed as means and standard errors of the mean. Unpaired two‐tailed Student's *t*‐test was used to compare two groups. One‐way ANOVA followed by Tukey's multiple comparison test was used to assess the difference between multiple groups with one independent variable. Two‐way ANOVA was used to compare multiple groups with two independent variables followed by Tukey's multiple comparison test. Statistical significance for the survival curve was determined by the log‐rank (Mantel–Cox) test. *P* < 0.05 was considered to be statistically significant.

## 3 RESULTS

### 3.1 *E. coli* SyBV are produced with higher yield and purity than OMV, and most cytosolic components are removed

**FIGURE 1:**
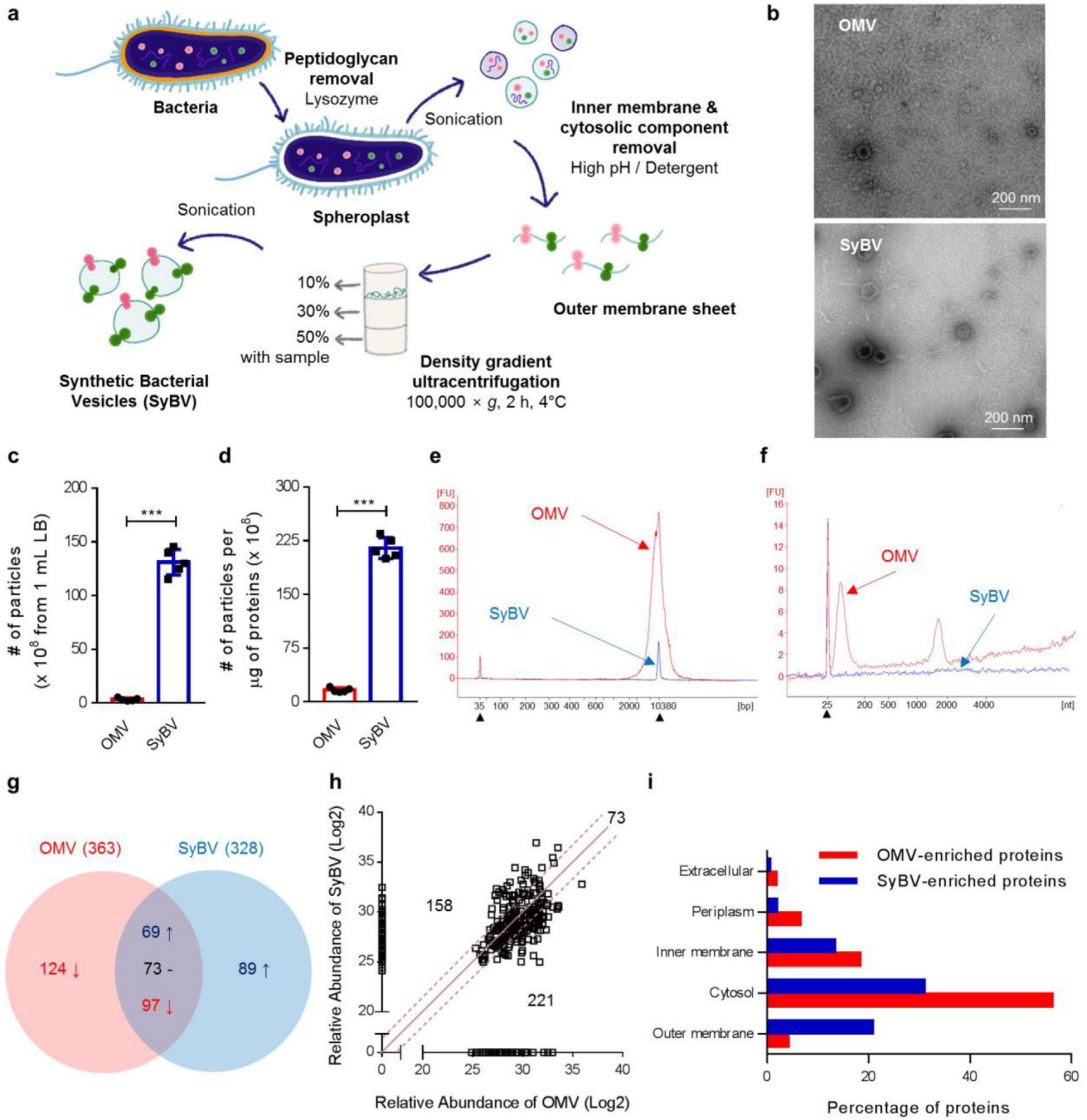
Production and characterization of *E. coli*‐derived SyBV. (a) Schematic diagram of the isolation of bacterial SyBV. (b) Representative TEM images of natural OMV and SyBV (three biological replicates for each sample and 10 pictures collected for each). Scale bars, 200 nm. (c, d) The number of particles derived from 1 ml culture media (c) and the number of particles per one microgram of vesicular proteins (d) from OMV and SyBV. Data are presented as the mean ± S.E.M. from five independent experiments. ^***^ *P* < 0.001 by unpaired two‐tailed Student's *t*‐test. (e, f) Representative electropherograms of DNA (e) and RNA (f) molecules isolated from SyBV in comparison to those from OMV. Three independent experiments, and filled triangles indicate internal markers. (g) Venn diagram of the OMV and SyBV proteomes. The common proteins are divided into three groups – a 1.5‐fold increase, a 1.5‐fold decrease, and no change – based on relative protein abundance. Two biological replicates were performed per sample, and three technical replicates were performed for each biological replicate. (h) Plot of the log2 value of relative protein abundance from OMV and SyBV. The solid line and dotted lines indicate no change and 1.5‐fold changes, respectively. (i) Proteomes of OMV‐enriched proteins and SyBV‐enriched proteins were analysed by GO cellular component annotations. Note that proteins normally have several GO annotations

*E. coli* SyBV were generated directly from bacterial pellets according to the procedure shown in Figure 1a. Briefly, *E. coli* cells were incubated with lysozyme to remove periplasmic components and then sonicated to disrupt the cell membranes. The resulting membranes were resuspended in the ionic detergent Sarkosyl to solubilize and remove the bacterial inner membrane, (Filip et al., 1973) and they were further treated with high pH to eliminate cytosolic components through the formation of membrane sheets. The clean membrane sheets were collected from the interface layer of 10% and 30% iodixanol after buoyant density‐gradient ultracentrifugation, and SyBV were obtained through mild sonication of the purified membrane sheets. Natural OMV were also produced from the *E. coli* culture supernatant and used for comparison with SyBV. TEM analysis of SyBV showed that they were closed spherical vesicles 50–150 nm in diameter, similar to OMV (Figure 1b and Figures S1, S2). However, unlike OMV, SyBV presented with a purer form without any pili‐like structures. *E. coli* SyBV were generated at a concentration 40 times higher than natural OMV from the same culture volume (Figure 1c), and they were 15 times purer in terms of the particle number per μg of protein, which was consistent with the TEM analysis (Figure 1d).

We next determined the compositional differences between SyBV and OMV because these might be associated with changes in the safety profile. Natural OMV presented with large DNA (Figure 1e) and RNA peaks (Figure 1f) according to bioanalyser analysis, whereas only a weak DNA peak was detected in SyBV, thus, showing that the genetic materials were almost completely removed in the process of SyBV generation. This is important because DNA or RNA derived from bacteria can be recognized by the nucleic acid‐recognizing TLRs on immune cells, resulting in excessive inflammation (Pandey et al., 2014). We next determined the protein composition of SyBV and OMV by comparative quantitative proteomics analysis. Principal component analysis indicated that the first component separated 55% of the data based on vesicle type (SyBV and OMV), and the second component separated 21% of the data by replicate (Figure S3a). Hierarchical cluster analysis showed similar results, with samples grouping first as the kind of vesicles and then by replicate (Figure S3b), demonstrating that there are unique protein profiles for each sample and that the biological replicates are closest together. In total, 363 and 328 proteins were identified by mass spectrometry from OMV and SyBV, respectively (Figure 1g). A total of 239 proteins were identified in both vesicle preparations, whereas 124 and 89 proteins were uniquely identified in OMV and SyBV, respectively (Table S1). Based on the relative protein abundance, 73 proteins did not change markedly in quantity among common proteins. However, 158 and 221 proteins were relatively increased and decreased in SyBV, respectively (Figure 1g, h). The subcellular localization of the enriched proteins in OMV and SyBV was examined by GO term analysis to compare cellular components (Figure 1i). In contrast to the OMV proteome, many cytosolic proteins were removed in the SyBV proteome. Also, SyBV were enriched with OMPs, which was consistent with the Coomassie brilliant blue staining of bacterial proteins (Figure S3c). In particular, OmpA, a major constituent of the outer membrane known to be involved in immune responses (Rainard et al., 2017), was detected in SyBV (Figure S3d). Moreover, the SyBV proteome was enriched in biological processes including transport and cellular response to damage, whereas the OMV proteome was shown to be more involved in protein translation (Figure S3e). Collectively, these findings suggest that our approach to the development of SyBV yields massive amounts of highly pure vesicles with few contaminating cytosolic proteins.

To further optimize the conditions for producing SyBV, SyBV were produced by different high pH conditions (pH 9–11) as shown in Figure S4a. There were no morphological differences in SyBV produced at different pH (Figure S4b), and the DNA was similarly reduced by all conditions according to the bioanalyser results (Figure S4c), thus, confirming the removal of cytoplasmic components under all conditions. However, pH 11 was the optimal condition for SyBV production in terms of yield and purity (Figure S4d, e), and this pH was used in all subsequent experiments.

### 3.2 *E. coli* SyBV do not induce any toxicity, even at a much higher dose than OMV

**FIGURE 2:**
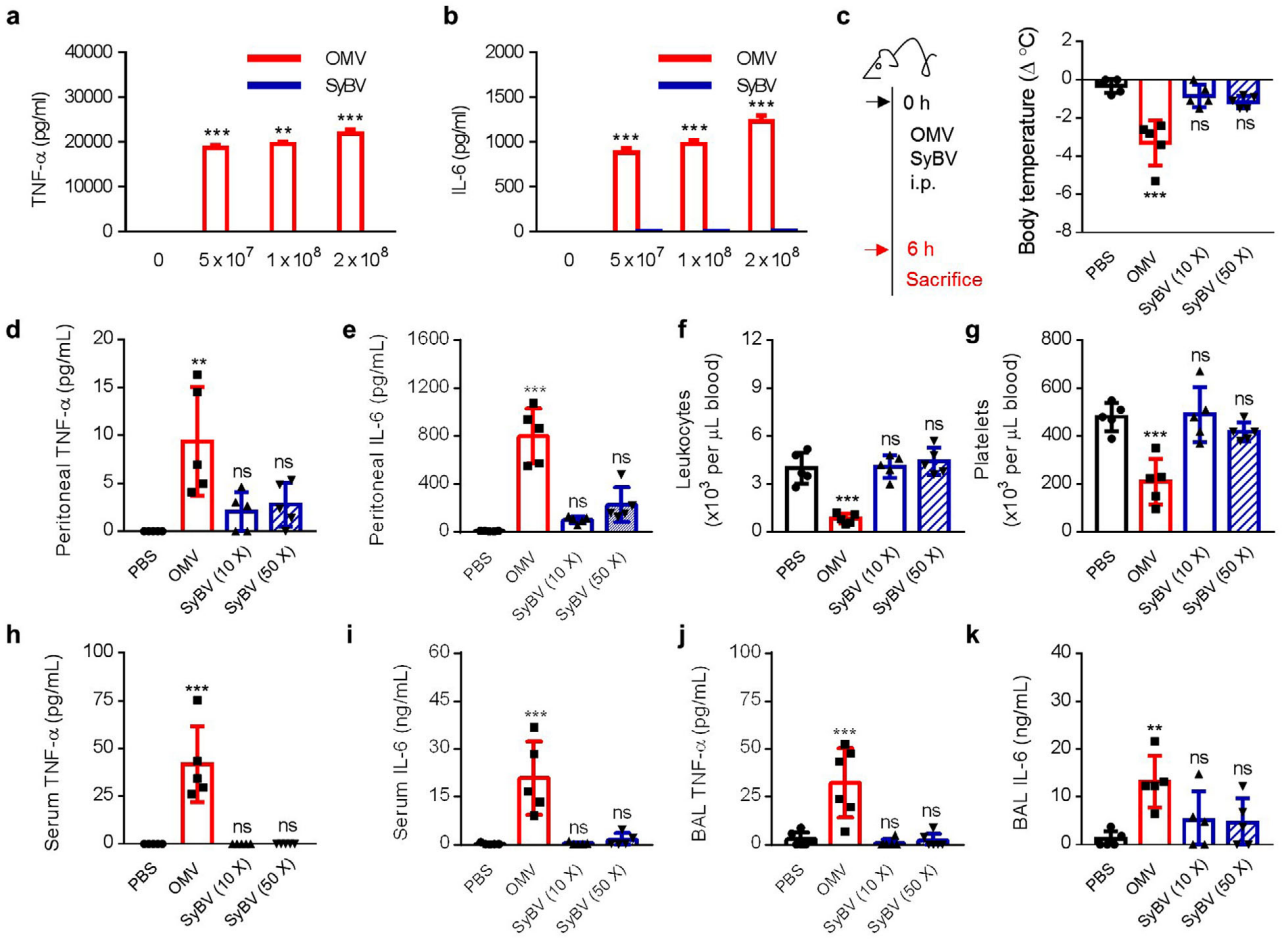
*E. coli* SyBV have no toxicity even at much higher doses than natural OMV. (a, b) Levels of pro‐inflammatory cytokine TNF‐α (a) and IL‐6 (b) production from RAW 264.7 cells treated with various doses of OMV or SyBV as indicated in the figure for 24 h (*n* = 3 independent samples). Two‐way ANOVA with Tukey's post test was used (vs. the ‘0’ group). (c) Study design for investigating the toxicity of SyBV in vivo (left panel). Mice were i.p. injected with OMV (5 × 10^9^) SyBV 10× (5 × 10^10^), or SyBV 50× (25 × 10^10^), and body temperature was measured at 6 h (right panel; *n* = 5 mice per group). (d, e) Peritoneal fluid cytokines such as TNF‐α (d) and IL‐6 (e) at 6 h (*n* = 5 mice per group). (f, g) The numbers of leukocytes (f) and platelets (g) in blood (*n* = 5 mice per group). (h, i) Serum level of TNF‐α (h) and IL‐6 (i) at 6 h (*n* = 5 mice per group). (j, k) The concentration of TNF‐α (j) and IL‐6 (k) measured in BAL fluid (*n* = 5 mice per group). Throughout, data are presented as mean ± S.E.M. ^**^ *P* < 0.01, ^***^ *P* < 0.001; ns, not significant, by one‐way ANOVA with Tukey's post test versus the PBS group (c–k)

To assess whether SyBV induce inflammatory toxicity *in vitro*, we used the macrophage cell line RAW 264.7, which is known to respond to bacterial components through the secretion of pro‐inflammatory cytokines (Sugitharini et al., 2013). The OMV induced a profound release of TNF‐α and IL‐6, whereas these were almost undetectable following treatment with SyBV (Figure 2a, b). To investigate the *in vivo* safety of SyBV, we used a mouse model that has a sepsis‐like response to OMV (Svennerholm et al., 2017). Mice were intraperitoneally (i.p.) injected with OMV or SyBV (at a 10‐fold or 50‐fold excess over OMV) after which various inflammatory profiles were evaluated in relation to local and systemic inflammation. No statistical differences in body temperature were observed in the SyBV‐treated groups versus non‐treated mice, while OMV provoked hypothermia, a commonly observed feature in this sepsis model (Granger et al., 2013) (Figure 2c).

Because intraperitoneal administration is related to acute and local inflammation in the peritoneum, we next assessed pro‐inflammatory cytokines in the peritoneal cavity. There was a non‐significant increase in TNF‐α (Figure 2d) and IL‐6 (Figure 2e) in the SyBV‐treated group, whereas OMV induced substantial inflammatory cytokine release. In terms of systemic inflammatory responses, SyBV treatment had no effect on the number of leukocytes (Figure 2f) or platelets (Figure 2g) in the blood. However, OMV significantly reduced the number of blood cells, which is considered to be caused by OMV‐induced immune activation and coagulation (Park et al., 2010). Moreover, we found that SyBV administration did not cause any increase in systemic cytokines in contrast to OMV (Figure 2h, i). Given our previous findings that OMV can exert a toxic effect on distant organs such as the lung (Park et al., 2010), we next evaluated whether SyBV have any inflammatory effects in the lung by measuring cytokines in BAL fluid. In accordance with the cytokine profiles in peritoneal fluid and blood, SyBV did not induce significant cytokine production in BAL fluid, as opposed to OMV (Figure 2j, k). Furthermore, to investigate which TLR signalling is related to the reduced toxicity of SyBV versus OMV, we compared stimulated TLR on engineered HEK‐293 cell lines in vitro. Specifically, SyBV activated TLRs 3, 7, 8 and 9 less than natural OMV did (Figure S5), which is consistent with our previous finding that the SyBV are devoid of RNA and DNA, agonists for TLRs 3, 7, 8 and 9 (Figure 1e, f).

Taken together, these results indicate that pure SyBV induce few toxic side effects even at much higher doses than OMV, making them a safer candidate for cancer immunotherapy.

### 3.3 *E. coli* SyBV are taken up by dendritic cells and enhance cell maturation through cytokine and surface molecule expression

**FIGURE 3:**
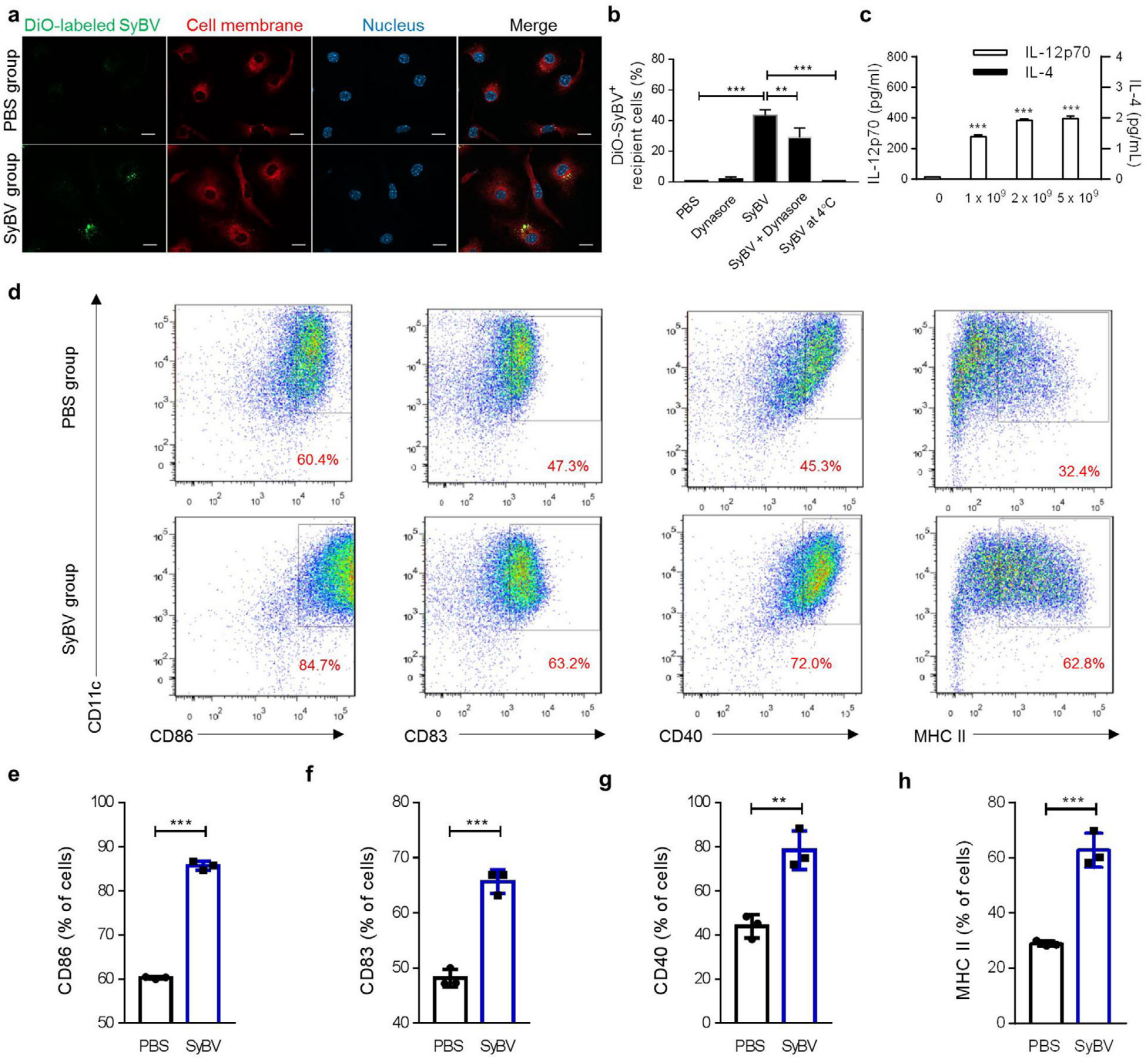
*E. coli* SyBV are taken up by dendritic cells and trigger maturation and activation of the cells. (a) Representative images of uptake of DiO‐labeled SyBV (green) by BMDCs at 6 h (two biological replicates and five pictures collected for each). Scale bars, 20 μm. (b) BMDCs were preincubated with dynasore for 1 h at 37°C, followed by treatment with DiO‐labeled SyBV for 6 h at 37˚C. SyBV were also incubated with the cells for 6 h at 4°C. The uptake of SyBV by cells was analyzed with flow cytometry, and data represent the percentage of DiO‐positive cells (*n* = 3 independent samples). One‐way ANOVA with Tukey's post test was used. (c) The production of IL‐12p70 and IL‐4 from BMDCs treated with various doses of SyBV as indicated in the figure for 24 h (*n* = 3 independent samples). Two‐way ANOVA with Tukey's post test was used (vs. the ‘0’ group). (d) The expression levels of dendritic cell maturation markers were analyzed by flow cytometry following incubation with SyBV (5 × 10^9^) for 24 h. The representative flow cytometry images were chosen from three independent samples. The number indicates the percentage of cells within the gate. (e–h) Percentage of CD86^+^ (e), CD83^+^ (f), CD40^+^ (g), and MHC II^+^ (h) BMDCs for all groups as indicated in the figure (*n* = 3 independent samples). Unpaired two‐tailed Student's *t*‐test was used. Throughout, data are presented as mean ± s.e.m. ^**^ *P* < 0.01, ^***^ *P* < 0.001

Dendritic cells (DCs) are the most powerful antigen‐presenting cells and are the first line of cells to initiate adaptive immune responses (Steinman & Hemmi, 2006). We used BMDCs to assess how SyBV affect DC activity. The BMDCs were incubated with DiO‐labelled SyBV following staining with the membrane‐specific stain Cellmask Deep Red. As shown in the confocal images in Figure 3a and Figure S6a, SyBV were observed in the vicinity of the cell membranes, and moreover, strong signals were observed intracellularly. However, treatment with free DiO‐dye did not elicit such signals (Figure S6b, c). We did not observe any significant difference of uptake between SyBV and OMV (Figure S6b). We also found increased SyBV uptake with prolonged treatment time (Figure S7a), which was similar to the results of the FACS analysis (Figure S7b). Moreover, the drug dynasore, which is an endocytosis inhibitor (Kirchhausen et al., 2008), significantly reduced the uptake of SyBV by BMDCs, and there was barely any passive uptake of SyBV at 4°C (Figure 3b).

To assess the ability of SyBV to activate BMDCs, the production of cytokines from BMDCs was measured following SyBV treatment. The SyBV induced the production of the immune‐modulating cytokine IL‐12p70 (a Th1‐polarizing cytokine) in a dose‐dependent manner (Figure 3c). However, there was no change in the level of IL‐4 (a representative Th2‐polarizing cytokine), suggesting that SyBV lead to a more polarized Th1 response, which is related to a cytotoxic cellular response (Abbas et al., 1996). To determine the capacity of SyBV to induce BMDC maturation, the expression levels of the surface DC markers were measured in the cells treated with SyBV (Figure 3d). BMDCs incubated with SyBV had elevated expression of CD86, CD83, CD40, and MHC II compared with the PBS‐treated group (Figure 3e‐h). To further elucidate the stimulated signalling pathway, we investigated whether SyBV together with tEV could activate the nuclear factor‐ĸB (NF‐ĸB) signalling pathway, which is known to be essential for the activation of DC‐derived cytokines such as IL‐12 (Liu et al., 2005). As a result, the vesicles efficiently induced phosphorylation of the p65 subunit of NF‐ĸB (Figure S8), implying that NF‐ĸB signalling may contribute to the immunoregulatory response observed in other experiments. Taken together, these findings show that SyBV are efficiently taken up by BMDCs and that SyBV activate the cells and induce Th1‐polarizing cytokine production and the expression of DC maturation markers.

### 3.4 Immunization with tEV plus SyBV slows melanoma tumour growth through metastatic inhibition and shows synergism with anti‐PD‐1 immunotherapy

**FIGURE 4:**
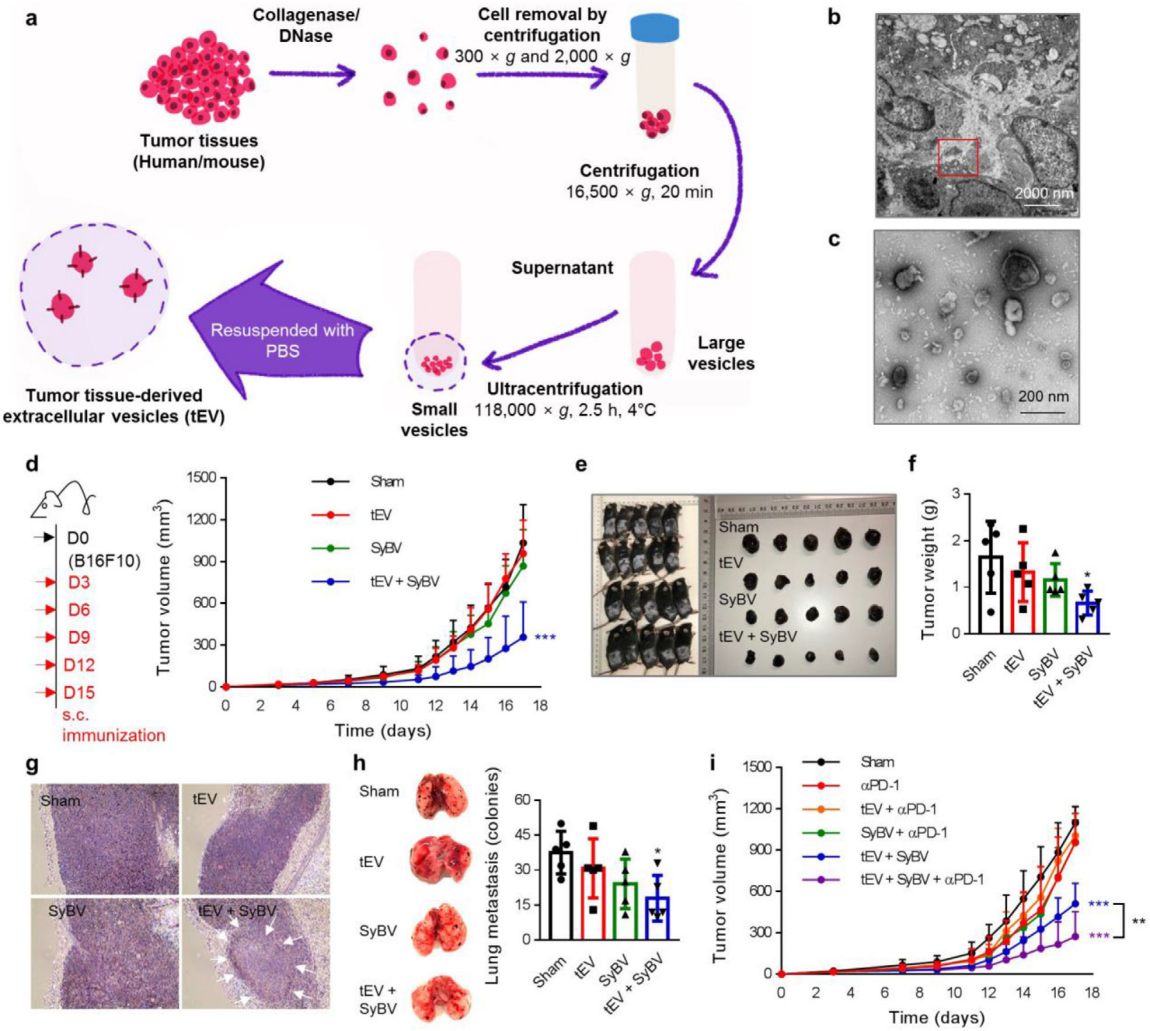
Therapeutic immunization with tEV plus SyBV inhibits melanoma growth and metastasis and shows a synergistic effect with anti‐PD‐1 immunotherapy. (a) Schematic diagram of isolation of tEV from melanoma tissue. (b) Representative TEM image of mouse melanoma tissue. The red box indicates tEV in the interstitial space of the tissues (two biological replicates and 10 pictures collected). Scale bar, 2000 nm. (c) Representative TEM image of isolated tEV (two biological replicates and 10 pictures collected). Scale bar, 200 nm. (d) Study design for therapeutic immunization (left panel). Mice were s.c. immunized with tEV (5 × 10^9^) and/or SyBV (5 × 10^9^) five times at 3‐day intervals following B16F10 inoculation. The tumour growth was monitored every 1 or 2 days (right panel; *n* = 14 from two independent experiments). (e, f) Pictures of mice and dissected tumours (e) and tumour weight (f) on day 17 after immunization (*n* = 5). (g) Representative melanoma histology images on day 17 after immunization (*n* = 5 and ten pictures collected for each). Arrows indicate necrotic areas at 10× magnification. (h) Representative photographs of pulmonary metastasis (left panel) and the number of pulmonary tumour nodules (right panel) on day 17 after immunization. (*n* = 5 and 10 pictures collected for each). (i) Tumour growth curve of mice immunized with tEV and/or SyBV plus anti‐PD‐1 therapy (*n* = 7). Throughout, data are presented as mean ± s.e.m. ^*^ *P* < 0.05, ^***^ *P* < 0.001 by one‐way ANOVA with Tukey's post test versus the sham group (d, f, h, i)

In this study, we chose to use tEV in order to induce specific anti‐tumour immune responses. We isolated tEV by treating tumour tissue pieces with DNase and collagenase, followed by sequential centrifugation and ultracentrifugation as shown in Figure 4a. Examination with TEM revealed the existence of vesicles in the interstitial space of mouse melanoma tissues (Figure 4b and Figure S9), and the purified tEV had nano‐sized structures with a closed spherical shape (Figure 4c). To address whether the therapeutic immunization with tEV plus SyBV blocks tumour growth, B16F10 cells were subcutaneously (s.c.) established in the flanks of mice, and tEV and SyBV were s.c. injected into the mice five times at 3‐day intervals once a palpable tumour had appeared. The combination of tEV and SyBV caused a significant reduction (65%) in the tumour volume, whereas sham, tEV alone, and SyBV alone did not show any inhibition (Figure 4d and Figure S10a‐d). Also, there was no effect by s.c. injection itself, and RAW 264.7‐cell derived vesicles did not show any beneficial effects (Figure S11). Moreover, there was a dramatic change in gross morphology in tumour‐bearing mice (Figure 4e) and tumour weights (Figure 4f) with increased necrotic areas (Figure 4g) in the tEV plus SyBV treatment group. The significant reduction in tumour growth led to increased survival, and mice immunized with tEV plus SyBV had a median survival time of 24 days compared to 18 days for the other groups (Figure S10e). Importantly, when using our immunization approach, no systemic toxicity was observed in the heart, liver, lung, or kidney (Figures S12 and S13), suggesting that SyBV enhance tumour vesicle‐driven anti‐tumour response without any severe toxicity. Co‐immunization of mouse colon tissue‐derived tEV and SyBV also showed inhibition of colon cancer growth, implying the extensive application prospects of this method in various cancers (Figure S14).

We next investigated the therapeutic effect of tEV plus SyBV on the growth of metastatic melanoma. Lung metastases were established by intravenous administration of B16F10 cells into mice, and then the mice were therapeutically immunized with tEV plus SyBV five times at 3‐day intervals. On day 17, the mice immunized with tEV plus SyBV showed significantly reduced numbers of melanoma lung colonies compared to the other groups (Figure 4h). Blocking PD‐1 expression on T cells has recently become the backbone of cancer immunotherapy because this activates T cells against the tumour (Chen & Han, 2015). Here, we further assessed the combined effect of anti‐PD‐1 treatment with tEV plus SyBV immunization. Briefly, mice were inoculated with B16F10 cells and then i.p. injected with αPD‐1 IgG 1 day prior to each immunization. The immunization with tEV plus SyBV together with αPD‐1 IgG therapy dramatically decreased tumour growth compared with αPD‐1 monotherapy and the tEV plus SyBV treatment groups (Figure 4i), indicating that the efficacy of tEV plus SyBV immunization could be boosted by anti‐PD‐1 inhibition in a synergistic manner.

### 3.5 Immunization with tEV plus SyBV stimulates tumour‐specific immunity characterized by Th1‐type humoral and cellular immune responses

**FIGURE 5:**
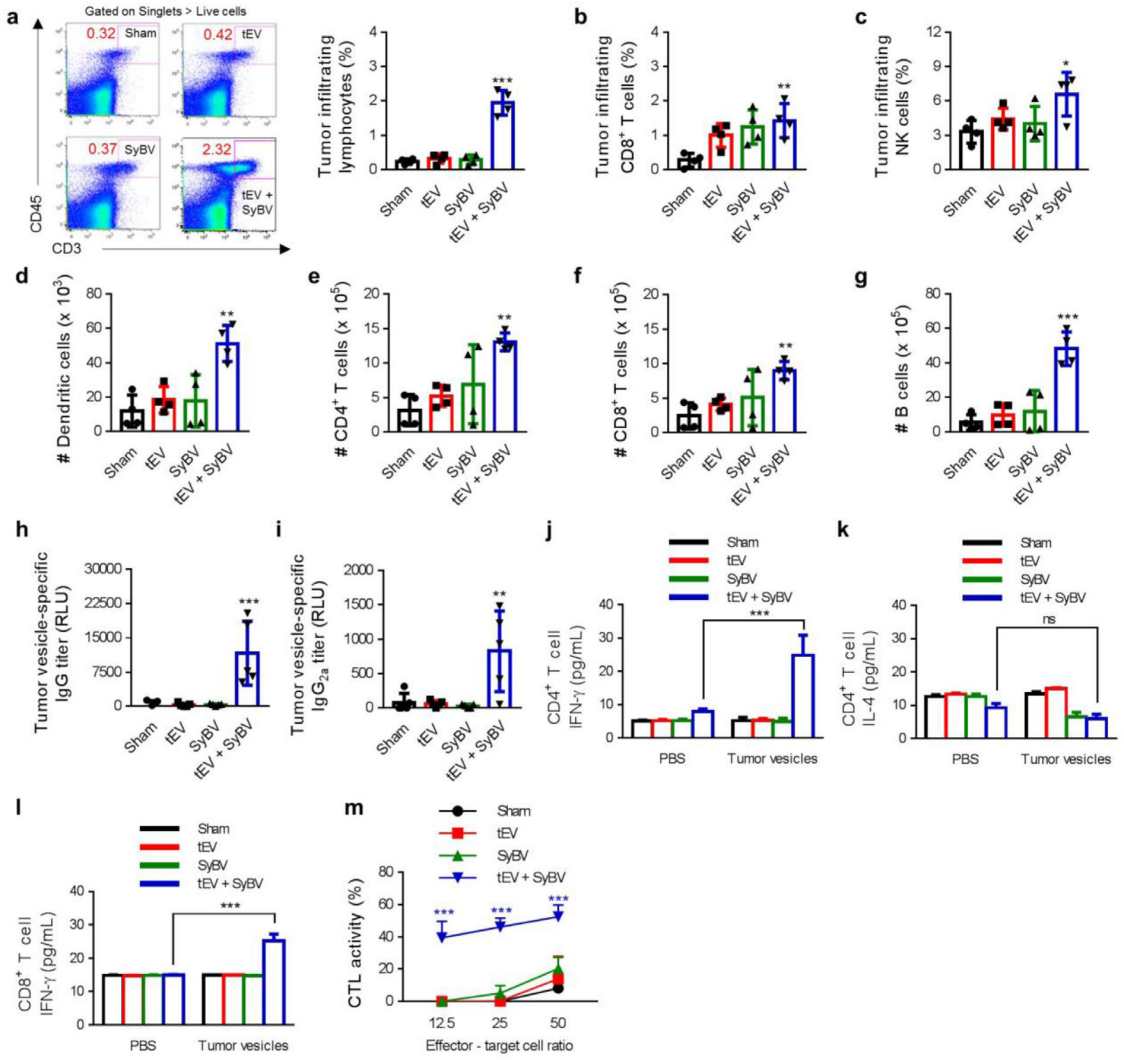
Immunization with tEV plus SyBV induces tumour‐specific cellular and humoral immunity, especially antitumour Th1/CTL responses. (a) Total tumour‐infiltrating lymphocytes were analyzed by flow cytometry on day 17 following immunization with tEV and/or SyBV. Representative flow cytometry images were chosen from four independent samples, and the number indicates the percentage of cells within the gate (left panel). The average percentages of cells are shown in the right panel. (b, c) Mean percentages of CD8^+^ T cells (b) and NK cells (c) infiltrating tumour tissues on day 17 from mice immunized with tEV and/or SyBV (*n* = 4). (d–g) Mean percentages of dendritic cells (d), CD4^+^ T cells (e), CD8^+^ T cells (f) and B cells (g) infiltrating inguinal lymph nodes on day 17 from mice immunized with tEV and/or SyBV (*n* = 4). (h, i) Tumour‐specific IgG (h) and IgG_2a_ (i) titers on day 17 from mice immunized with tEV and/or SyBV (*n* = 5). (j–l) The level of tumour‐specific CD4^+^ T cell‐derived IFN‐γ (j) and IL‐4 (k) and CD8^+^ T cell‐derived IFN‐γ (l) after CD4^+^ T cells and CD8^+^ T cells were isolated from immunized spleens (three independent samples). (m) Tumour‐specific CTL responses of splenic T cells prepared from immunized spleens (three independent samples). Throughout, data are presented as mean ± s.e.m. ^*^ *P* < 0.05, ^**^ *P* < 0.01, ^***^ *P* < 0.001; ns, not significant; by one‐way ANOVA with Tukey's post test versus the sham group (a‐i); by two‐way ANOVA with Tukey's post test versus the sham group (j–m)

To explore the mechanism by which tEV plus SyBV influence the anti‐tumour response, tumours were collected on day 17 following repeated immunization and tumour infiltrating lymphocyte populations were measured by flow cytometry. Immunization with tEV plus SyBV led to a significant increase in lymphocyte infiltration compared to the other groups (Figure 5a), and especially CD8^+^ T cells (Figure 5b) and natural killer (NK) cells (Figure 5c) in tumour tissue were highly infiltrated in mice immunized with tEV plus SyBV, and this correlated with the inhibition of tumour growth in this group (Figure 4d). We next evaluated the infiltrating immune cell profile by draining the lymph nodes, which are a primary cite for the initiation of effector immune responses (Shu et al., 2006). The number of DCs was significantly increased by immunization with tEV plus SyBV, and no change was observed in the sham, tEV alone, or SyBV alone groups (Figure 5d). In terms of the T cell and B cell composition within lymph nodes, CD4^+^ T cells (Figure 5e), CD8^+^ T cells (Figure 5f), and B cells (Figure 5g) were significantly increased in the mice immunized with tEV plus SyBV, implying the induction of tumour‐specific adaptive immunity by SyBV.

To further characterize the anti‐tumour adaptive immune response to our immunization protocol, we next assessed the humoral response by measuring tumour antigen‐specific antibody levels. Immunization with tEV plus SyBV increased the total amount of tumour‐specific IgG antibodies compared to sham, tEV alone, and SyBV alone (Figure 5h). Notably, tumour‐specific antibody isotyping revealed a significant increase in the Th1‐related IgG_2a_ antibodies in mice immunized with tEV plus SyBV (Figure 5i), indicating the development of a potent Th1‐type humoral immune response against melanoma. Given that a tumour‐specific cellular immune response is a key factor in cancer immunotherapy (Vermaelen, 2019), we next evaluated the tumour‐specific T cell responses by checking the production of key cytokines from splenic T cells. Re‐stimulation of isolated splenic CD4^+^ T cells with tumour antigens dramatically induced the secretion of the Th1 cytokine IFN‐γ in the group immunized with tEV plus SyBV (Figure 5j). However, there were no changes in the level of the Th2 cytokine IL‐4 in any of the groups (Figure 5k), suggesting that immunization with SyBV primes a tEV‐driven tumour‐specific Th1 immune response. CD4^+^ T cells have been shown to undergo polarization into Th1 cells in order to help with the production of anti‐tumour effector cells, namely CTLs (Kennedy & Celis, 2008). A significant increase in IFN‐γ from splenic CD8^+^ T cells, which is associated with CTL activity (Ghanekar et al., 2001), was seen in cells re‐stimulated with tumour antigens (Figure 5l). We then examined tumour‐specific CTL responses using B16F10 cells as the target cells. Differentiated splenocytes from mice immunized with tEV plus SyBV elicited a greater CTL response against B16F10 cells than splenocytes from mice immunized with sham, tEV alone, or SyBV alone (Figure 5m). Taken together, our results show that SyBV together with tEV promoted a balanced humoral and cellular response to tumour antigen, and this was accompanied by polarization of Th1/CTL subtypes.

### 3.6 The SyBV have greater immunoadjuvant activity in human tEV‐specific immunity compared to other commercial adjuvants

**FIGURE 6:**
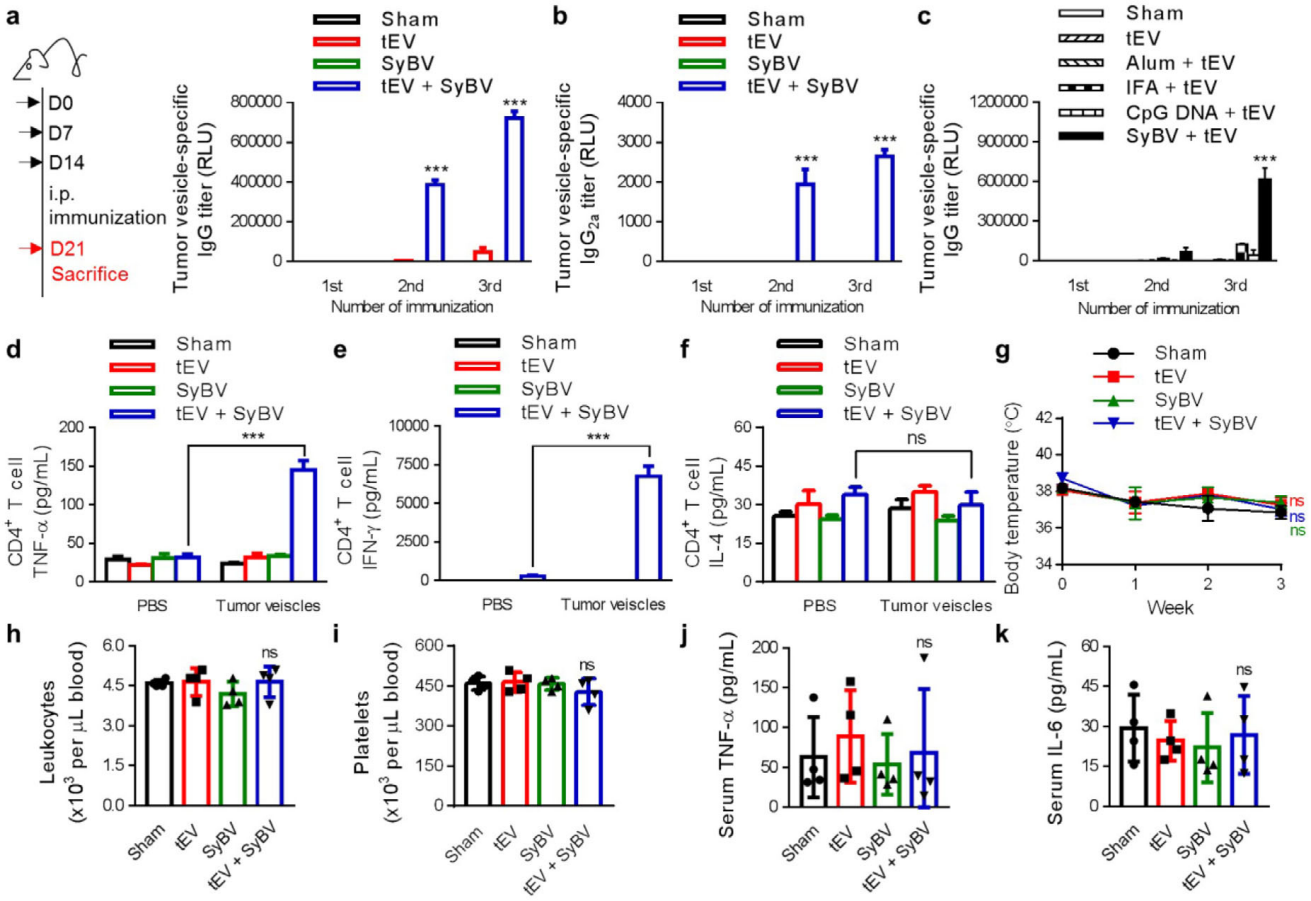
Human tEV‐specific immunity is stimulated by SyBV to a greater extent than other commercial adjuvants, whereas human tEV alone do not affect the immunity. (a) Study design for the evaluation of the immunogenicity of human tEV and SyBV (left panel). Mice were i.p. injected with human tEV (5 × 10^9^) and/or SyBV (5 × 10^9^) three times at weekly intervals, and then the human tEV‐specific IgG titre was measured in the blood (right panel; *n* = 5). (b) The level of human tEV‐specific IgG_2a_ titer in the blood from mice immunized with human tEV and/or SyBV (*n* = 5). (c) Comparison of the adjuvant activity of SyBV to that of other traditional adjuvants in terms of induction of human tEV‐specific IgG (*n* = 5). (d–f) The level of human tEV‐specific CD4^+^ T cell‐derived TNF‐α (d), IFN‐γ (e), and IL‐4 (f) after CD4^+^ T cells were isolated from immunized spleens (three independent samples). g, Body temperature measured over the course of immunization with human tEV and/or SyBV (*n* = 4). (h‐k) The number of leukocytes (h) and platelets (i) and the level of TNF‐α (j) and IL‐6 (k) measured in the blood on day 7 after immunization with human tEV and/or SyBV for 3 weeks (*n* = 4). Throughout, data are presented as mean ± s.e.m. ^***^ *P* < 0.001; ns, not significant; by two‐way ANOVA with Tukey's post test versus the sham group (a–g); by one‐way ANOVA with Tukey's post test versus the sham group (h–k)

To explore whether the findings from our immunotherapeutic studies with SyBV could be applied to human tumours, we first isolated tEV from melanoma tissues obtained from patients with lymph node metastasis using the isolation method described in Figure 4a. During immunization with human tEV and/or SyBV, serum was acquired from immunized mice weekly and the human tEV‐specific reactive IgG titre was measured (Figure 6a). Antibody titers were initially increased at the second immunization with human tEV plus SyBV and peaked after the third immunization. However, human tEV alone failed to significantly induce IgG production compared to sham mice, suggesting that human tumour vesicular proteins do not elicit strong immunogenicity without the assistance of SyBV. Moreover, immunization of mice with human tEV plus SyBV increased Th1‐related IgG_2a_ production compared with sham, human tEV alone, and SyBV alone (Figure 6b), which corresponds to the findings in mice immunized with mouse tEV plus SyBV (Figure 5i).

Given that many kinds of adjuvants have been developed for cancer immunotherapy, including the most commonly used alum and IFA (Temizoz et al., 2016), we compared the immunoadjuvant activity of SyBV with other traditional adjuvants. The highest antibody response against human tEV was obtained by immunization with SyBV compared to alum, IFA, and the TLR agonist CpG DNA (Figure 6c), implying that the adjuvant effect of SyBV is the most efficient for the development of tumour‐specific immunity. Next, we assessed human tEV‐specific T cell responses induced by SyBV. The human tEV‐specific production of TNF‐α (Figure 6d) and IFN‐γ (Figure 6e), the key cytokines produced by Th1 cells, was significantly increased after the last immunization with human tEV plus SyBV. However, Th2 cytokine IL‐4 production was similar in all immunized groups (Figure 6f), which is consistent with our findings in mice immunized with tEV plus SyBV (Figure 5j, k). Furthermore, there were no changes in body temperature (Figure 6g) or evident systemic effects on blood cells (Figure 6h, i) during immunization with human tEV plus SyBV. Also, the immunization had no effect on systemic pro‐inflammatory cytokines in blood (Figure 6j, k). Collectively, these results suggest that SyBV can be safely and efficiently exploited for human tumour‐specific immunotherapy and that they are a more suitable adjuvant than other candidates.

## 4 DISCUSSION

In this study, bacterial SyBV were explored as a novel adjuvant platform to determine their immunotherapeutic potential in developing humoral and cellular immunity, leading to potent anti‐tumour effects. Recently, natural OMV have gained increased attention as the next generation vaccine carrier due to their highly immunogenic nature, their ability to target lymph nodes, their easily modified genome, and the ability to load heterologous antigens (Collins, 2011; Gerritzen et al., 2017). However, the associated studies are still in their infancy, especially regarding OMV applications in cancer research. Despite various advantages of OMV as an effective cancer adjuvant, they carry pro‐inflammatory components such as LPS and other virulence factors that have the capacity to activate innate immunity through interaction with TLRs (Pathirana & Kaparakis‐Liaskos, 2016). It has been reported that *E. coli* OMV provoke a sepsis‐like inflammatory response, activate endothelial cells and platelets related to a procoagulant state, and induce further cardiac dysfunction (Park et al., 2010; Soult et al., 2014; Svennerholm et al., 2017). To overcome these toxicities, we have proposed a strategy to generate purified artificial vesicles from bacteria through chemical modification with lysozyme and detergents. We conducted the SyBV isolation steps under high pH (Figure 1a), which causes the formation of membrane sheets (Go et al., 2019) that can be separated from the cytosolic components. These membrane sheets can then be returned to spherical structures by the addition of energy, and here we used mild sonication. The artificially produced SyBV showed similar morphology and diameter as natural OMV (Figure 1b), but contained very few contaminants and had no or very little RNA or DNA inside (Figure 1d‐f). Our proteomic analysis further confirmed that SyBV carry relatively fewer cytosolic proteins compared to OMV (Figure 1i), suggesting that our approach using high pH is the optimal way to solve the safety issues with natural OMV by removing cytosolic components and possibly detoxifying membrane glycoproteins.

Cancer immunotherapy is considered to be a cost‐effective method for treating cancer diseases. However, there is a need for improvement in cancer vaccines, especially in terms of adjuvants that are sufficiently potent to boost a tumour‐specific immune response (Banday et al., 2015). To develop the most suitable adjuvant for cancer vaccines, several important aspects should be considered, including safety and efficacy. As shown in Figure S5, in contrast to OMV, SyBV could not activate TLR 3, 7, 8 and 9, which would have been activated by bacteria DNA and RNA, which obviously are lacking in the SyBV. These TLR receptors are known to contribute to pro‐inflammatory innate cytokine production in bacterial infection. Moreover, they can have a cross‐talk with TLR 2 and 4, leading to synergistically massive cytokine production (Tan et al., 2014), in part explaining why SyBV did not cause any toxicity when given in much higher doses than OMV, both *in vitro* and *in vivo* (Figure 2). In terms of efficacy, SyBV were shown to be easily internalized into BMDCs and could efficiently stimulate DC maturation (Figure 3), revealing high potency in stimulating an adaptive immune response. It is generally thought that natural OMV interact with target cells via multiple internalization pathways such as micropinocytosis, endocytosis, and fusion, although there are still substantial discrepancies between studies (O'donoghue & Krachler, 2016). Here we elucidated the endocytic internalization of SyBV into BMDCs using dynasore, an inhibitor of dynamin (Figure 3b). Dynamin is a GTPase involved in clathrin‐mediated endocytosis, and inhibition of the protein disrupts vesicle internalization in target cells (Feng et al., 2010). Similarly, dynasore inhibited the entry of SyBV into cells, indicating the contribution of clathrin‐mediated uptake, although the relative importance of other uptake pathways remains to be explored.

It has been reported that the gut microbiome, including the Ruminococcaceae family, can modulate anti‐tumour response to checkpoint inhibitor (Gopalakrishnan et al., 2018). However, no study has yet investigated whether these probiotic bacteria produce vesicles related to anti‐tumour effect, and whether those vesicles are available to the immune system to regulate the anti‐tumour immune response. Because OMV contain a variety of immunomodulatory proteins for activating antigen‐specific immunity, OMV‐based immunotherapeutic studies have been well described in infectious diseases as well as in cancer (Kim et al., 2013; Kim et al., 2017; Wang et al., 2017). Kim *et al*. have recently reported that bacterial OMV given intravenously are capable to induce colon cancer regression in mice (Kim et al., 2017). However, OMV can induce cytokine storm via TLR cross‐talk, and it is unclear whether LPS‐mutation is sufficient to reduce such severe side effects. Another advantage with SyBV versus OMV is that bacteria usually release relatively smaller amounts of OMV in the culture supernatants, making the production of SyBV potentially more cost‐inefficient. The low yield of OMV is also attributed to time‐consuming procedures such as filtration and concentration of a large volume of culture medium. In order to avoid this low productivity issue, SyBV were directly generated from bacterial membranes, allowing a smaller bacterial culture to produce larger numbers of vesicles. Importantly, SyBV were purified with a 40‐fold higher yield than natural OMV from same culture volume (Figure 1c), suggesting the economic benefit of SyBV as a cancer vaccine adjuvant. In our efficacy studies, we did not directly compare the immunotherapeutic effects of SyBV and OMV. However, it is expected that they have comparable potencies in stimulating the immune system because they share bioactive vesicular OMPs such as OmpA (Figure S3c, d).

Current cancer vaccine approaches, including both peptide‐based and DNA‐based vaccines, require DNA sequencing of the individual patient and the manufacturing of tumour‐specific antigens for that patient (Lu & Robbins, 2016). These drawbacks can be overcome using whole‐cell tumour vaccine made from tumour tissue acquired during surgery, which is an easily available method for cancer immunotherapy without laborious sequencing and antigen synthesis. However, immunotherapy using tumour cell lysate‐based vaccine is relatively inefficient and fails to elicit a strong CTL response and only leads to weak tumour regression (De Gruijl et al., 2008; Sondak & Sosman, 2003). Importantly, cancer immunotherapy with tumour cell‐derived extracellular vesicles has gained increased interest in the scientific community (Taghikhani et al., 2020). This approach was found to be more beneficial for triggering enhanced immunogenic response versus soluble tumour cell lysates due to prolonged presence of tumour antigens in addition to greater uptake by DCs (Joshi et al., 2014). Gu *et al*. have previously reported that tumour EV‐based vaccines can be superiour to tumour lysates in tumour‐bearing mice. Therapeutic vaccination with EV prolonged the survival time of mice, and more DCs and T cells were significantly recruited into the tumour compared to tumour lysate‐based vaccine (Gu et al., 2015). Our group has recently developed a new technique to directly isolate extracellular vesicles from melanoma tissues (tEV) without tumour cell culture (Figure 4a) (Crescitelli et al., 2020; Crescitelli et al., 2021; Jang et al., 2019). Also, in line with our findings (Figure 4b, c), it was confirmed that tEV are nanosized structures enriched with melanoma membrane proteins, suggesting that these vesicles are an ideal source of tumour antigens to achieve potent therapeutic vaccine effects. It is not guaranteed that tEV do not harbour any contaminants from other cellular components. However, we gently sliced the tumour tissues and exposed them to enzymes that can dissociate interstitial components, and are known to not affect cell structure and EV marker proteins (Autengruber et al., 2012; Crescitelli et al., 2020). Importantly, no aggressive vortexing or tissue homogenization was not used, which potentially would avoid the presence of cellular debris in the tEV isolates. Also, iodixanol‐based cushion ultracentrifugation was performed to separate pure vesicles from aggregated soluble proteins. Thus, it is expected that natural and clean vesicles can be produced from the tumour isolates although we cannot exclude the possibility of some contaminants caused by the experimental procedure. Also, our group has previously investigated various populations of cells included in the tumour tissues as determined by flow cytometry (Crescitelli et al., 2020). The majority of large cells included tumour cells mixed with small populations of neutrophils, macrophages and eosinophils, indicating that most isolated vesicles might be coming from tumour cells although the relative portion of other cells‐derived vesicles remains to be further explored. As shown in Figures 4 and 5, tEV themselves had low immunogenicity and therapeutic efficacy and therefore they require the use of appropriate adjuvants to give a strong immune response. In this study, we used an immunization strategy combining tEV and SyBV to elicit anti‐tumour immunity. We found that co‐administration of tEV and SyBV suppressed melanoma growth significantly (Figure 4d), whereas immunization with tEV alone did not result in any slowing of tumour growth, strongly suggesting that the presence of SyBV affords high immunogenicity to tEV‐derived antigens.

Although anti‐PD‐1 therapy has shown promising clinical outcomes, there is a still hurdle because only a limited number of patients respond. Importantly, checkpoint inhibition therapy is dependent on the patient's endogenous tumour‐specific CTL responses, and sometimes this is used in combination with other conventional therapeutics to attain optimal efficacy (Park & Cheung, 2017). Thus, we investigated the effect of combining αPD‐1 IgG therapy and immunization with tEV plus SyBV. Treatment with tEV plus SyBV in combination with an αPD‐1 antibody synergistically inhibited melanoma growth, whereas there was no therapeutic effect in the αPD‐1 monotherapy group (Figure 4i), suggesting that therapeutic immunization with tEV plus SyBV is a useful strategy for augmenting the anti‐tumour effect of anti‐PD‐1 therapy. We next investigated the underlying mechanism by which the combined immunization of tEV and SyBV might work in inhibiting cancer. Co‐injection of SyBV with tEV induced high concentrations of tEV‐antigen‐specific IgG antibodies, specifically Th1‐related IgG_2a_ (Figure 5h, i). Also, the level of the Th1 cytokine IFN‐γ released by CD4^+^ T cells in the spleen was enhanced after immunization with tEV plus SyBV (Figure 5j), which was also found by Morishita et al. using engineered tumour extracellular vesicles with CpG DNA for melanoma regression (Morishita et al., 2016). Furthermore, tEV plus SyBV treatment stimulated tEV antigen‐specific CTL responses with an increase in IFN‐γ (Figure 5l, m), which is considered to be more essential than the role of CD4^+^ T cells for the substantive killing of tumour cells (Farhood et al., 2019). Collectively, our findings concerning immune mechanisms suggest that SyBV contribute to the Th1‐biased immune responses against tEV antigens and are linked to enhanced CTL responses for efficient anti‐tumour immune responses.

As mentioned above, CTLs are critical components for cancer immunotherapy, but the difficulty in activating tumour‐specific CTL responses has been recognized as one of the main hurdles in the development of cancer vaccine adjuvants (Banday et al., 2015). Although alum is commonly used as a licensed vaccine adjuvant for human use, it fails to induce robust Th1 or CTL responses (Pashine et al., 2005). Several studies have reported that the generation of sustainable CTL responses can be achieved through the combination of other TLR ligands such as Poly I:C and CpG DNA (Temizoz et al., 2016). Given that bacterial outer membranes are composed of various immune modulators that target TLRs (Lee et al., 2016), outer membrane‐derived SyBV are expected to synergistically induce potent immunogenicity. Notably, the effect of SyBV on the induction of tumour antigen‐specific IgG is more potent than that of alum, IFA, and CpG DNA (Figure 6c), which suggests that SyBV are an optimal combination of complexes containing powerful immunostimulants. Another important aspect for a successful cancer adjuvant is whether it is effective and tolerated for human use. To test whether SyBV can potentially trigger immunogenicity against human tumour antigens, we measured human tEV‐specific IgG from mice immunized with SyBV together with tEV derived from human melanoma. The results confirmed that the involvement of SyBV is critical for inducing a human tumour antigen‐specific antibody response (Figure 6a). Moreover, administration of human tEV and SyBV did not induce systemic inflammation (Figure 6g‐k), indicating that our immunization approach might be safely applied in human cancer immunotherapy.

Our study demonstrates for the first time the immunotherapeutic effect of isolated SyBV from bacteria on melanoma growth when combined with tumour tEV, and it shows the role of humoral and cellular immune responses in specific anti‐tumour immunity. Moreover, our data suggest that SyBV might be cost‐effective because of their high yield and stronger efficacy compared to other conventional adjuvants, and they appear to be non‐toxic and thus suitable for individualized treatment of different cancers, specifically to increase the proportion of patients who respond to checkpoint inhibitors. Further research is needed to determine the duration of the anti‐tumour immune response with our novel approach, and whether combining the SyBV with other experimental immuno‐oncology tools could increase efficacy further.

## CONFLICTS OF INTEREST

Jan Lötvall and Kyong‐Su Park have filed multiple patents for the development of mammalian and bacterial vesicles as therapeutics. Jan Lötvall owns equity in Codiak BioSciences Inc. and Exocure BioSciences Inc. Kyong‐Su Park is financed by Exocure Biosciences Inc. The other authors have no disclosures related to this work.

## AUTHOR CONTRIBUTIONS

Kyong‐Su Park designed the study, performed and analyzed the experiments, interpreted the results, and wrote the manuscript. Kristina Svennerholm was involved in ethical approval for the mouse study and wrote the manuscript. Rossella Crescitelli helped collect tumour vesicles from human tumour samples and wrote the manuscript. Cecilia Lässer performed the TEM analysis and wrote the manuscript. Inta Gribonika performed and analyzed the FACS experiments and the animal study. Jan Lötvall conceived the study, supervised the design, interpreted the results, and wrote the manuscript.

## Supporting information

Supporting information.
