# Supplementary material for: Synthetic bacterial vesicles combined with tumour extracellular vesicles as cancer immunotherapy
Source: J Extracell Vesicles. 2021 Jul 3;10(9):e12120. doi: 10.1002/jev2.12120 (PMC8254025; doi:10.1002/jev2.12120)
Supplement: Supplementary file 1 — Supporting information. [file JEV2-10-e12120-s001.docx]

**
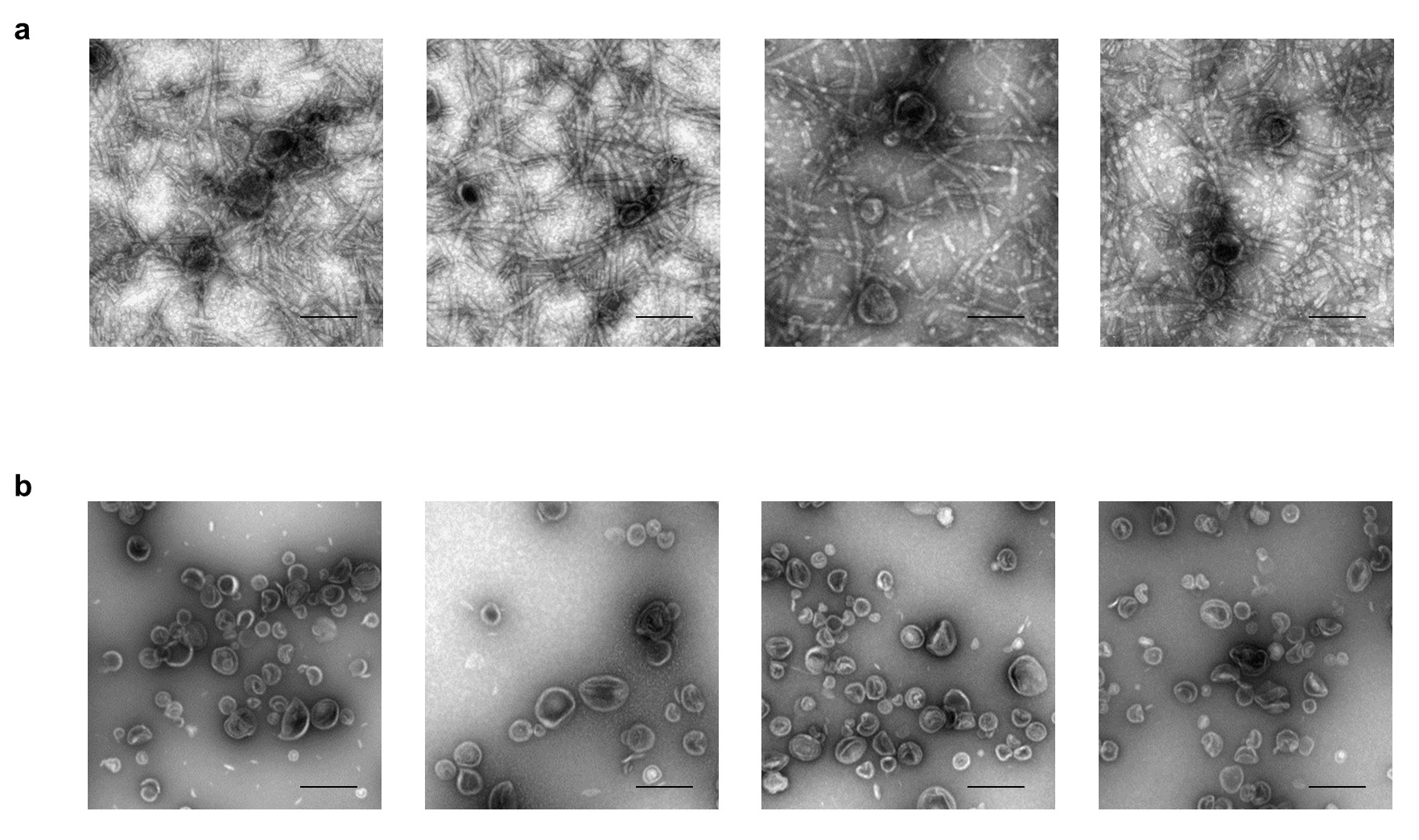
**

**Supplementary Figure 1. Morphological characterization of *E. coli*-derived OMV and SyBV. a**,**b**, TEM images of natural OMV (**a**) and SyBV (**b**). Scale bars, 200 nm.

**
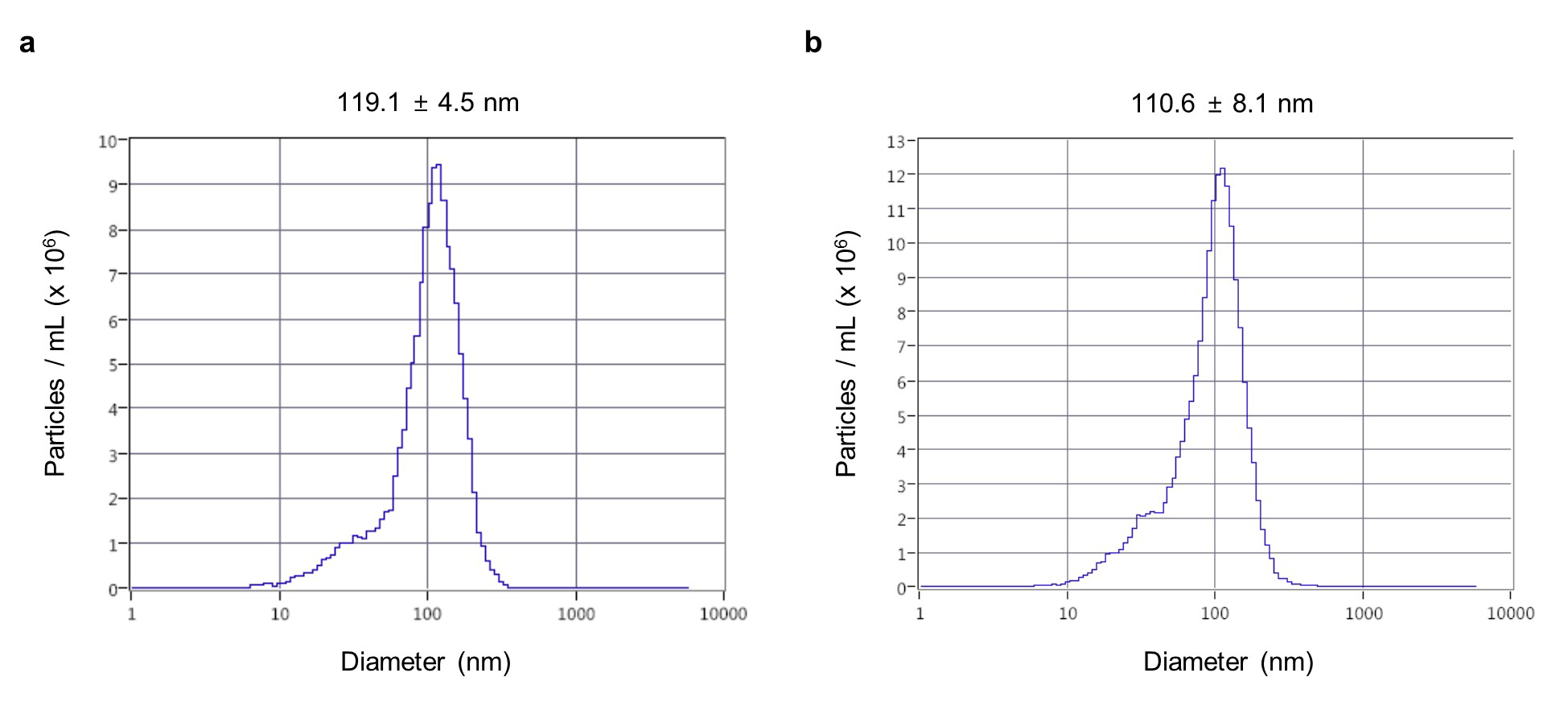
**

**Supplementary Figure 2. Size distribution of *E. coli*-derived OMV and SyBV. a**,**b**, The size of OMV (**a**) and SyBV (**b**) were measured by nanoparticle tracking analysis with triplicate samples.

**
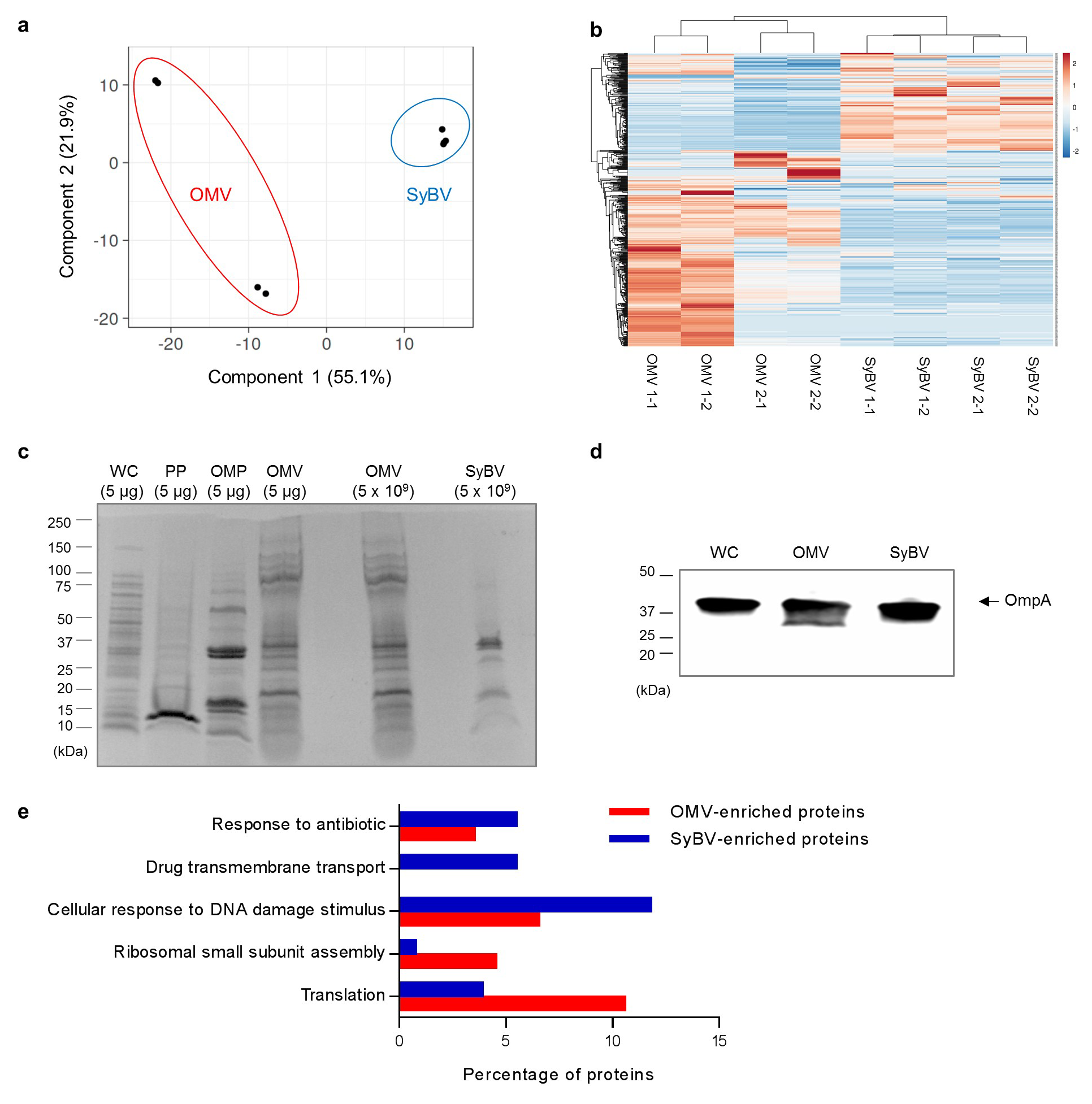
Supplementary Figure 3. Comparison of protein composition between natural OMV and SyBV. a**, Principle component analysis of OMV and SyBV proteome. Two biological replicates per sample and three technical replicates for each biological replicate. **b**, Partial view of heatmap of the hierarchical clustering based on relative proteins abundance of each proteome. Color code shows the normalized median abundance of proteins belonging to the category (red, most abundant; blue, least abundant). **c**, Coomassie brilliant blue staining of bacterial whole-cell lysates (WC), outer membrane proteins (OMP), periplasmic proteins (PP), OMV and SyBV. This is the whole image of the gel. **d**, Western blot analysis of WC (10 µg), OMV (1 × 10^9^) and SyBV (1 × 10^9^) with anti-OmpA antibody. **e**, Proteomes of OMV-enriched proteins and SyBV-enriched proteins were analyzed by GO biological process annotations. Note that proteins normally have several GO annotations.

**
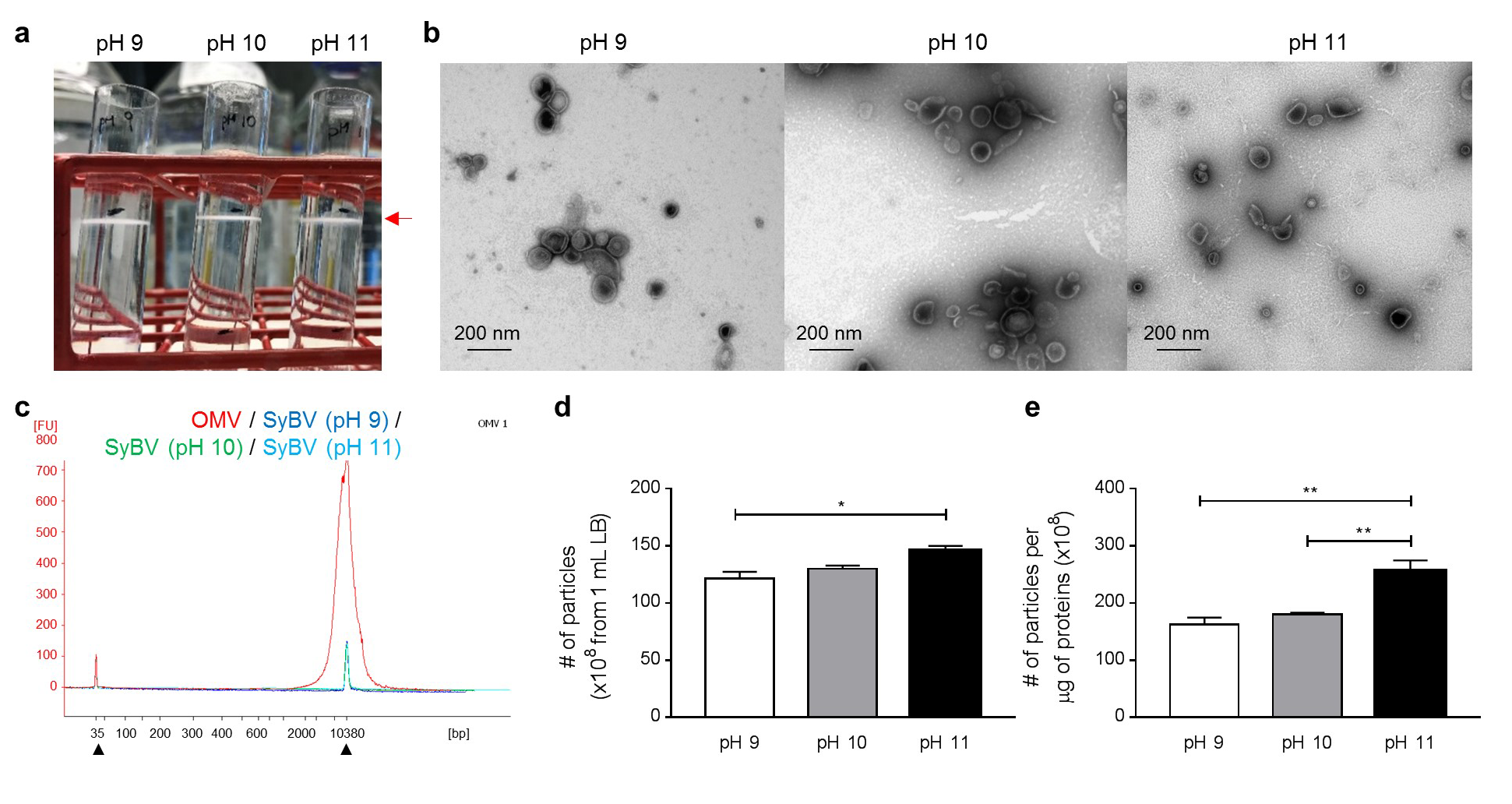
Supplementary Figure 4. Effect of different high pH on SyBV characters. a**, Photograph of SyBV treated with various high pH conditions followed by iodixanol gradient ultracentrifugation. Red arrow indicates SyBV in the layer between 10% and 30% iodixanol. **b**, Representative TEM images of SyBV isolated under different high pH (one biological replicate for each sample and ten pictures collected for each). Scale bars, 200 nm. **c**, Representative electropherogram of DNA molecules isolated from SyBV treated with various high pH. Three independent experiments, and filled triangles indicate internal markers. **d**,**e**, The number of particles derived from 1 mL culture media (**d**) and the number of particles per one microgram of vesicular proteins (**e**) from various high pH-treated SyBV. Three independent experiments and ^*^*P* < 0.05, ^**^*P* < 0.01 by One-way ANOVA with Tukey’s post test. Data are presented as mean ± s.e.m.

**
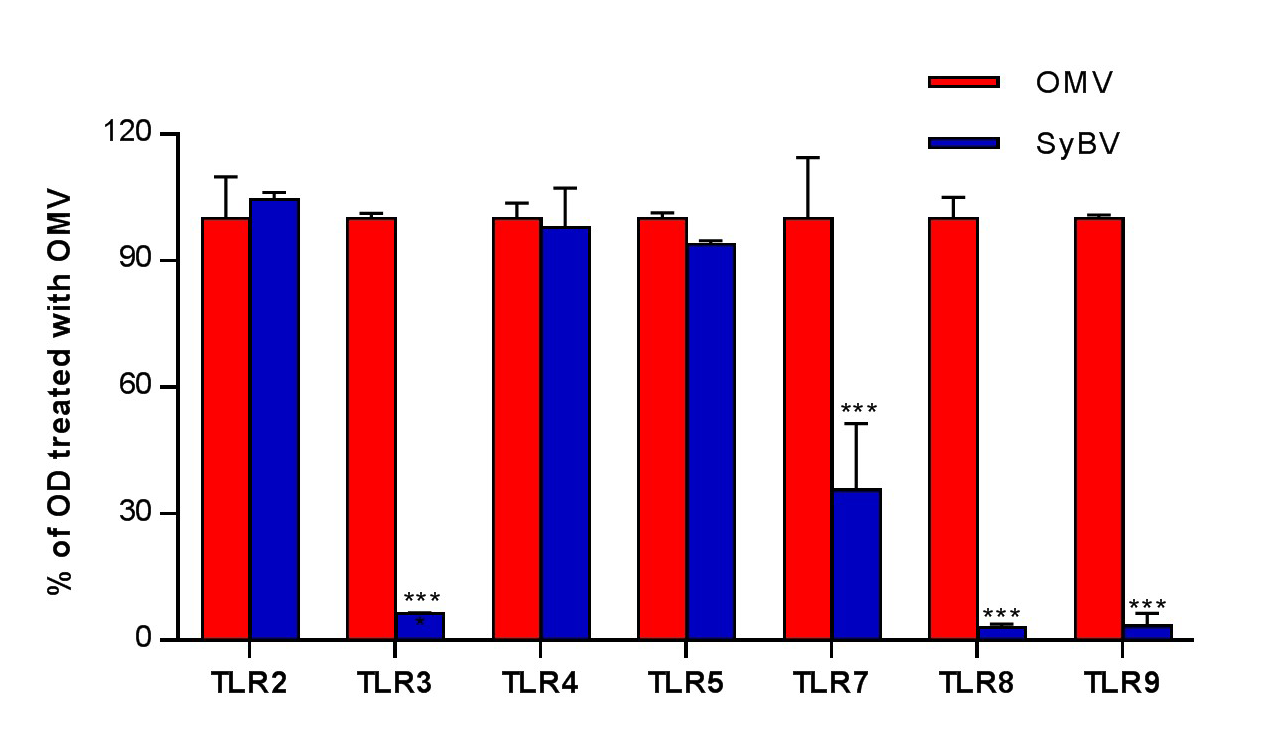
**

**Supplementary Figure 5. The contribution of SyBV to TLR signaling.** OMV or SyBV were treated to HEK-293 cell lines expressing TLR and NF-ĸB reporter gene for 18 h (*n* = 2 independent samples), and then optical density (OD) value was measured. Data were indicated as percentage of OD of OMV-treated cells. ^***^*P* < 0.001 by Two-way ANOVA with Tukey’s post test versus the OMV group. Data are presented as mean ± s.e.m.

**
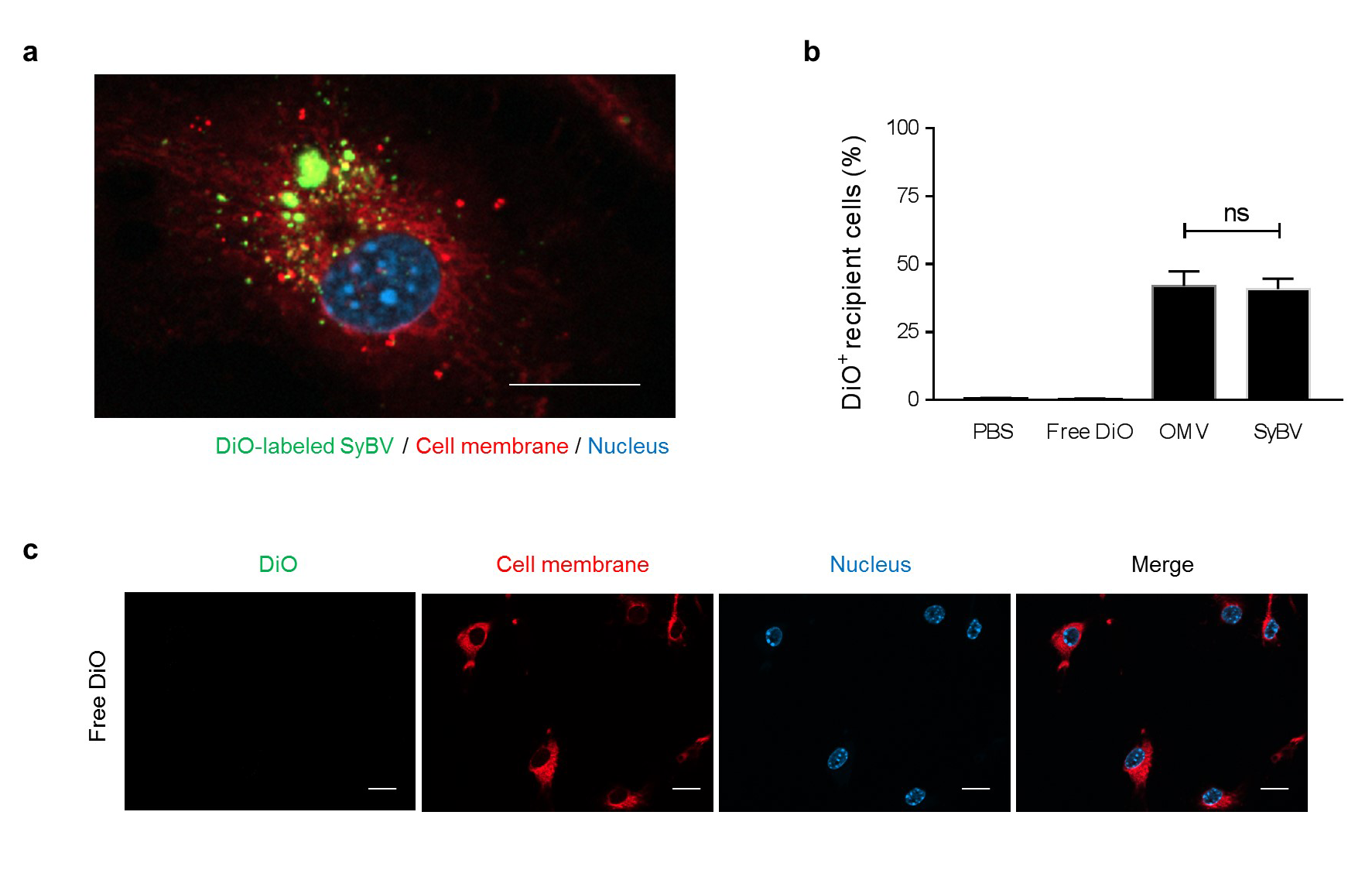
**

**Supplementary Figure 6. The comparison of uptake of SyBV with OMV. a**, The enlarged image which is six times as big as the original one shown in **Fig. 3a**. Scale bar, 20 µm. **b**, BMDCs were treated with free DiO, DiO-labeled OMV, and DiO-labeled SyBV for 6 h at 37˚C. And then, the uptake of vesicles was analyzed with flow cytometry, which is indicated by the percentage of DiO-positive cells (*n* = 3). Data are presented as mean ± s.e.m. ns, not significant by one-way ANOVA with Tukey’s post test. **c**, Representative images of BMDCs treated with free DiO at 6 h. Scale bars, 20 µm.


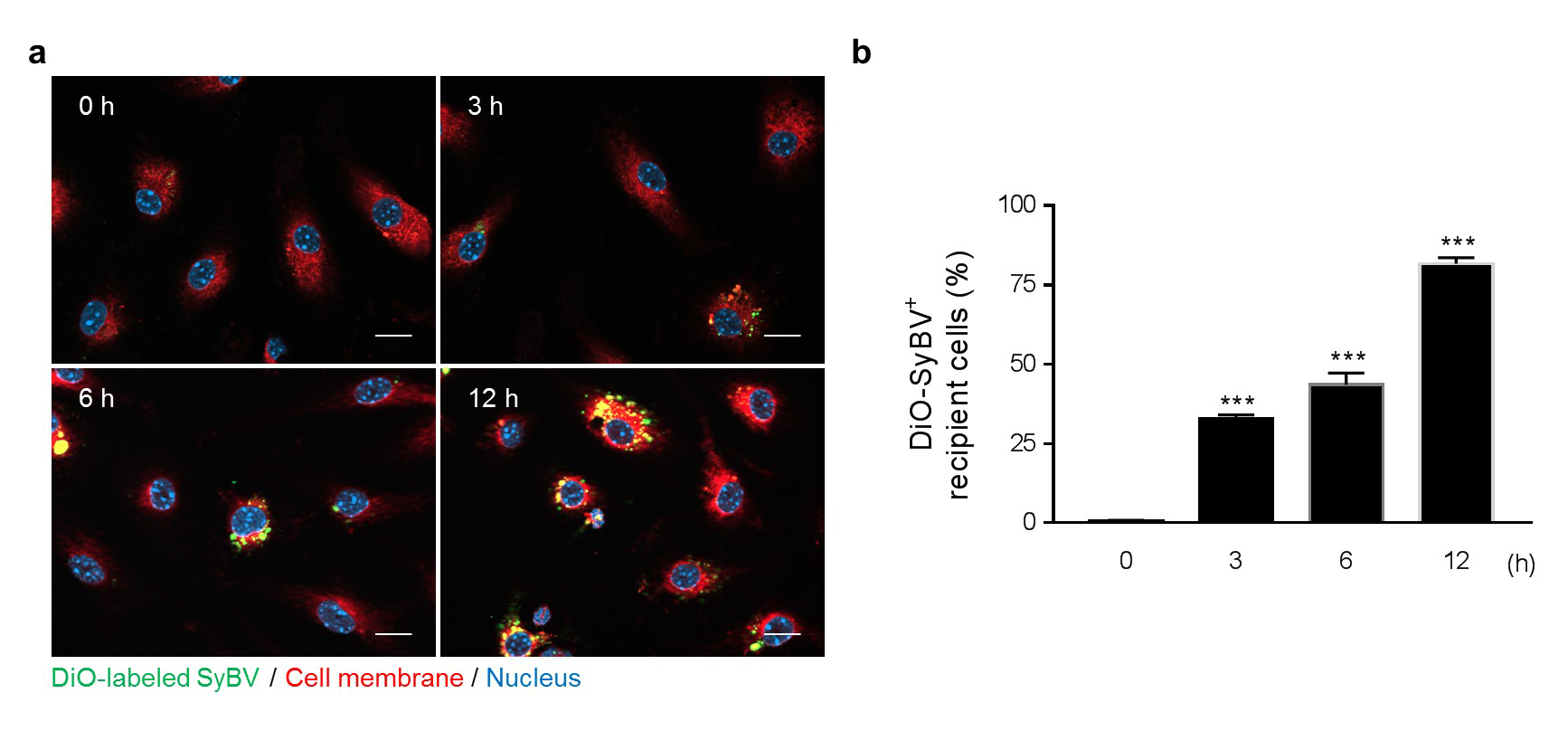


**Supplementary Figure 7. Time-dependent uptake of SyBV by BMDCs. a**, Representative images of uptake of DiO-labelled SyBV (green) by BMDCs stained with Cellmask Deep Red (red) and DAPI (blue) at different time points as indicated in the figure. (two biological replicates and five pictures collected for each). Scale bars, 20 µm. **b**, The uptake of the labelled SyBV by BMDCs was analyzed with flow cytometry, and data show the percentage of DiO-positive cells (*n* = 3 independent samples). Data are presented as mean ± s.e.m. ^***^*P* < 0.001; by one-way ANOVA with Tukey’s post test versus 0 group.

**
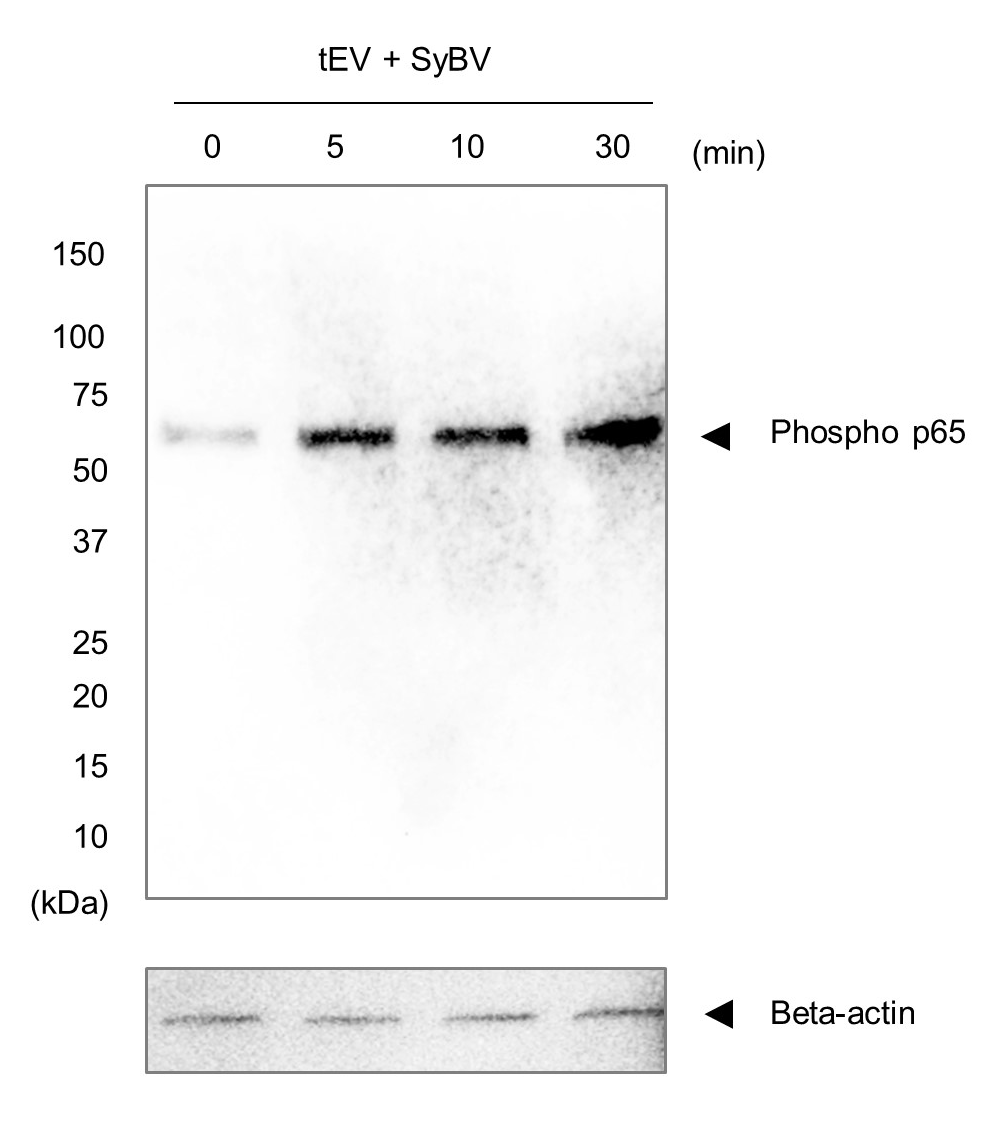
**

**Supplementary Figure 8. The effect of tEV and SyBV on the phosphorylation of p65 in BMDCs.** Cells were treated with tEV (5 x 10^9^) and SyBV (5 x 10^9^) for 0 - 30 min. The whole-cell lysates (30 µg) were probed with anti-phospho-p65 (serine 536) antibody (1/1000 dilution). For loading control, the blots were stripped and reprobed with anti-beta-actin antibody (1/1000). The images were cropped from different parts of the same gel.

**
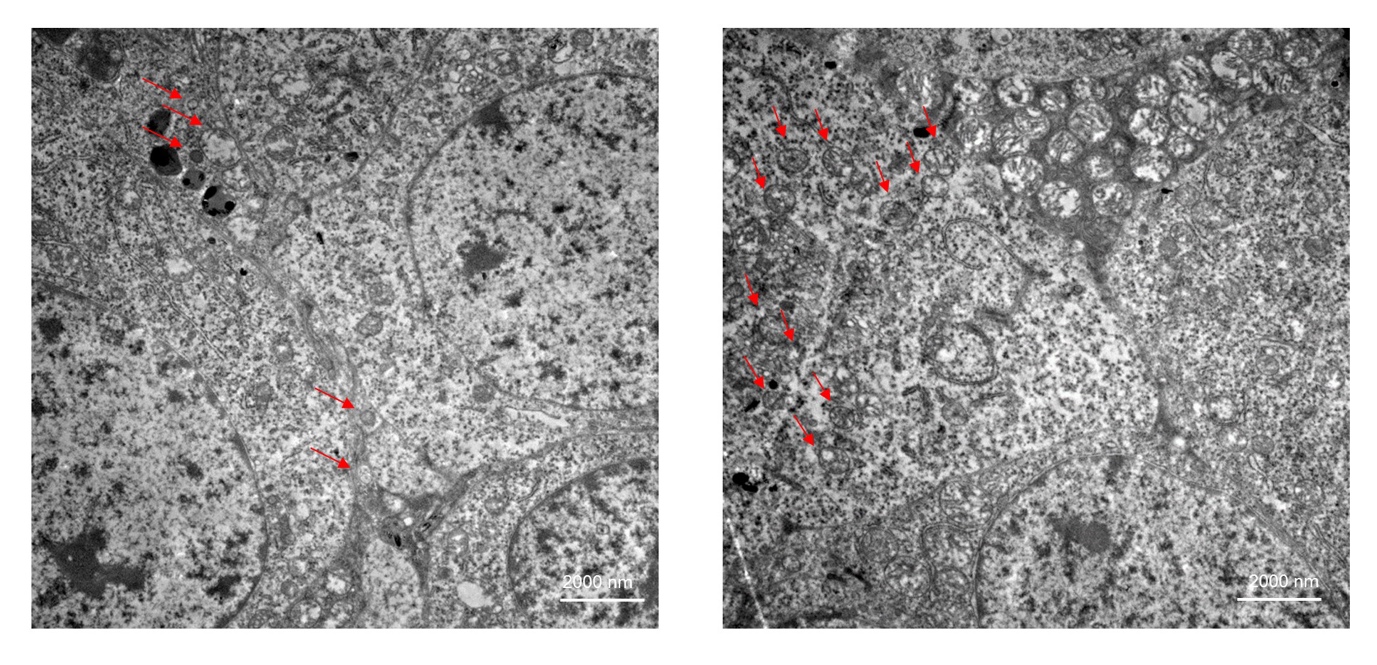
**

**Supplementary Figure 9. The TEM images of mouse melanoma tissue section.** The EV in extracellular space are indicated by red arrows. Scale bars, 2000 nm.


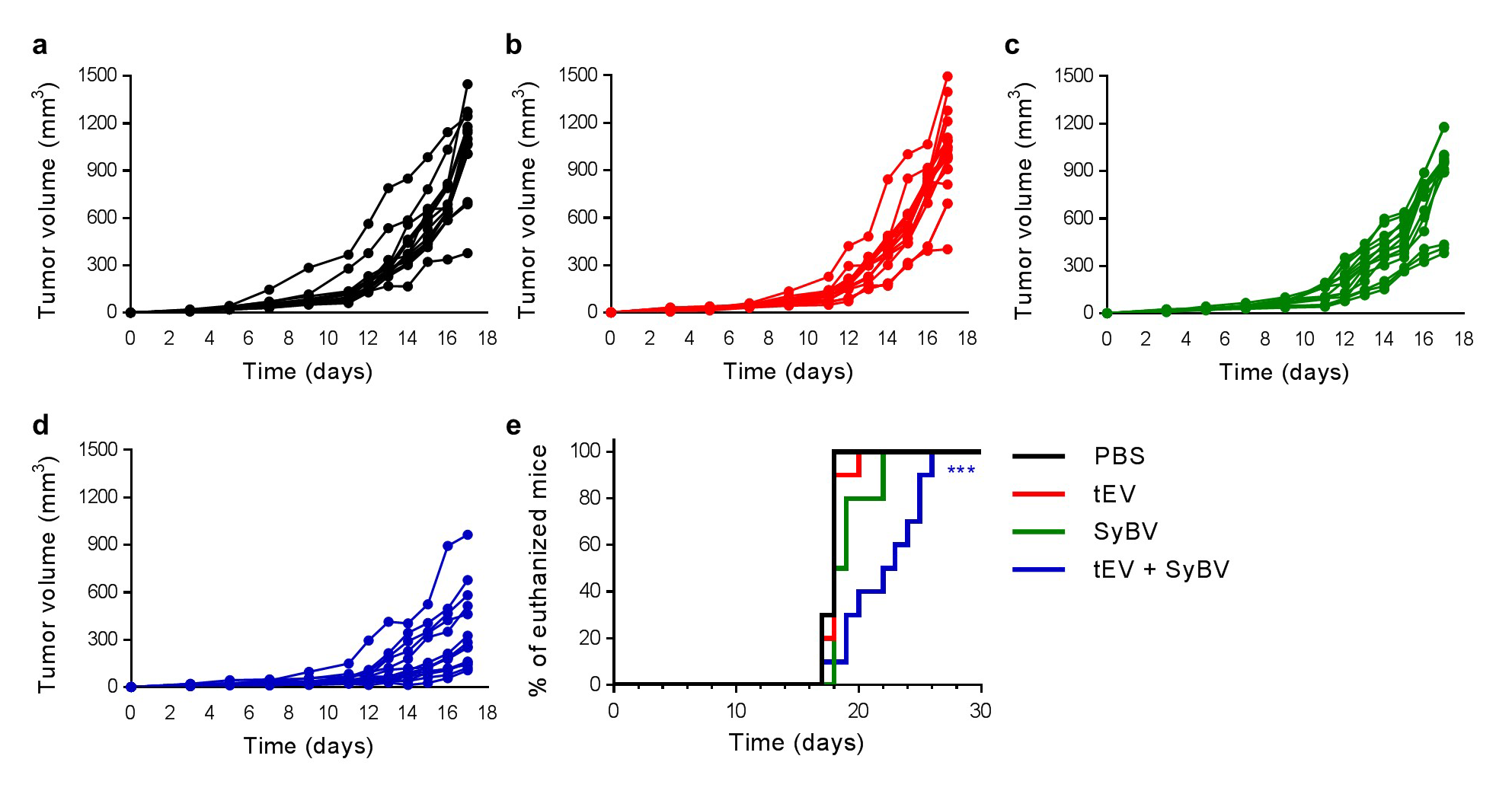


**Supplementary Figure 10. Therapeutic treatment with tEV and SyBV in B16F10 tumor-bearing mice. a**-**d**, Each individual tumor growth curve (**a**, sham; **b**, tEV; **c**, SyBV; **d**, tEV + SyBV) of mice shown in **Fig. 4d**. **e**, Overall percentage of euthanized mice after mice were immunized with tEV and/or SyBV (*n* = 14 mice per group from two independent experiments). ^***^*P* < 0.001; by log-rank (Mantel-Cox) test versus sham group.


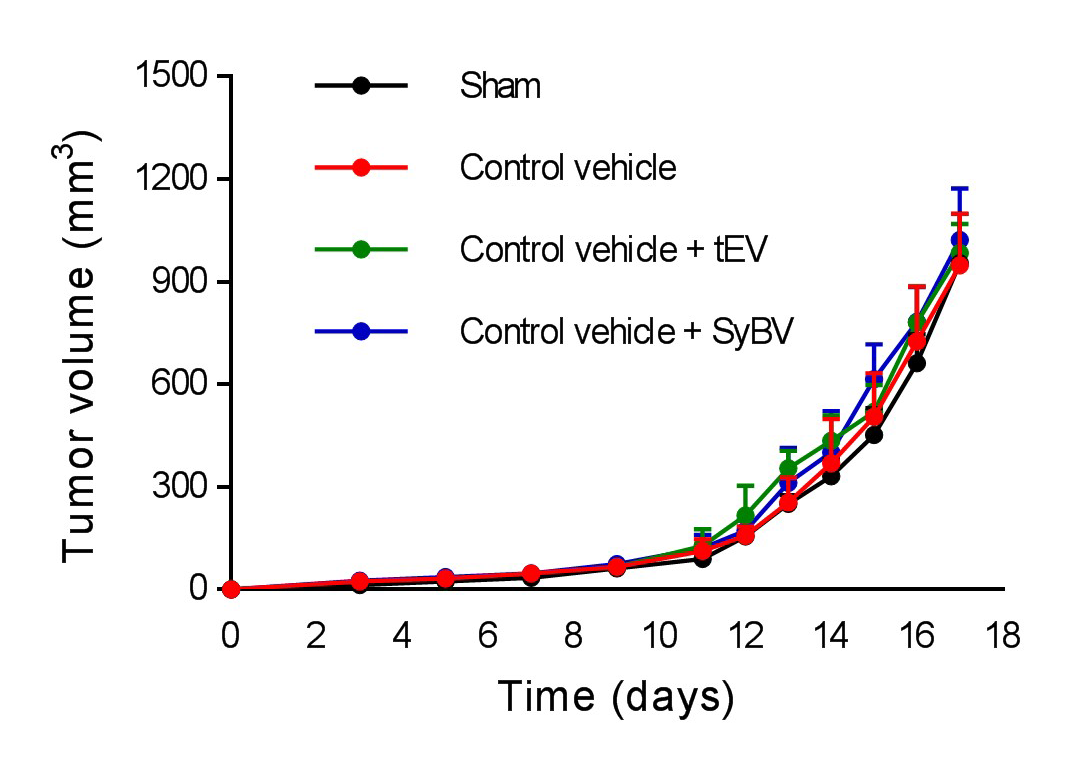


**Supplementary Figure 11. No effect of immunization with control vehicle in melanoma-bearing mice.** Extracellular vesicles from RAW 264.7 cells were used as control vehicle, and then mice were s.c. immunized with control vehicle alone (5 × 10^9^) or together with tEV (5 × 10^9^) or SyBV (5 × 10^9^) five times at 3-day intervals following B16F10 inoculation (*n* = 5). Data are presented as mean ± s.e.m.


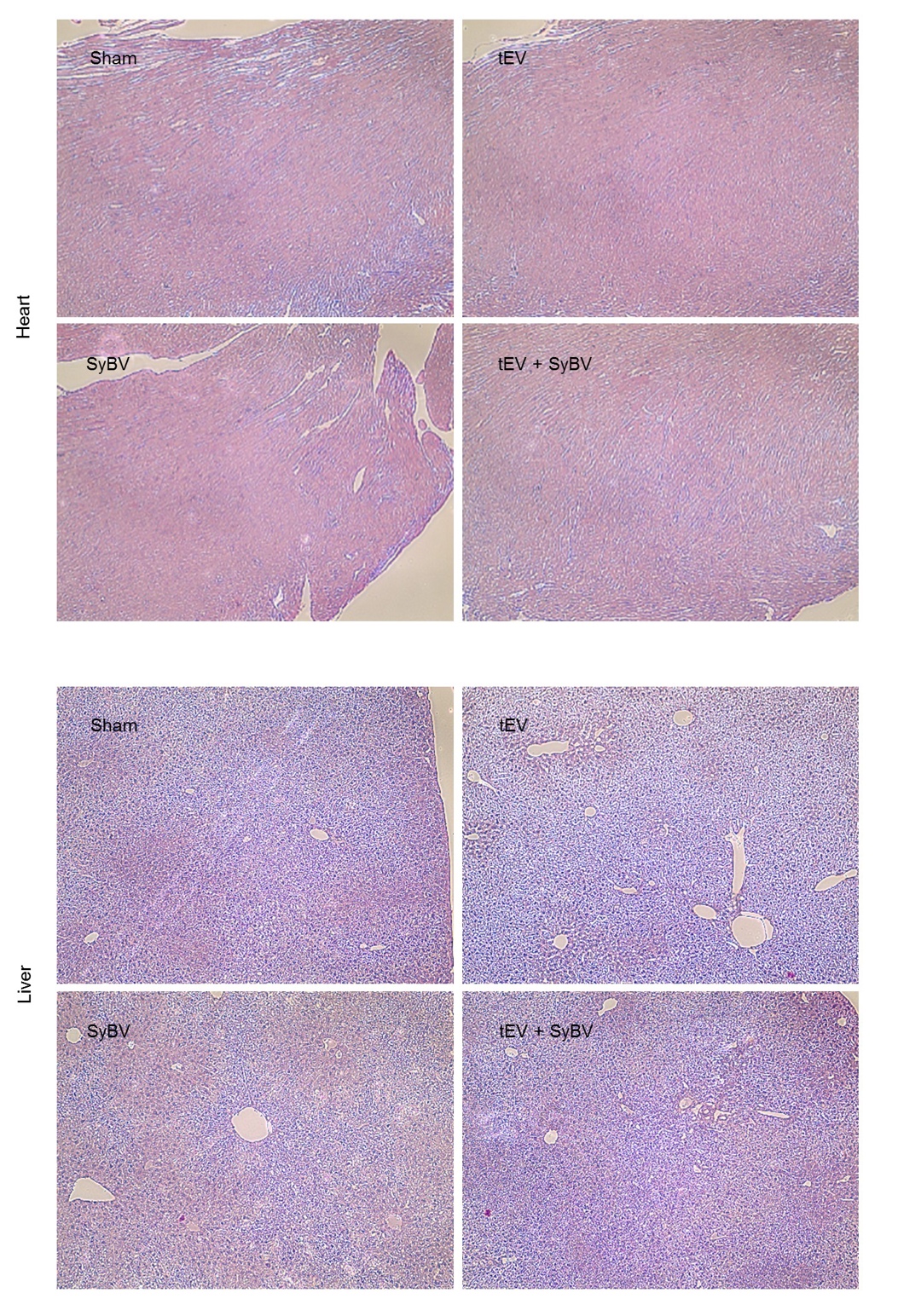


**Supplementary Figure 12. Safety profiles of mice immunized with tEV and SyBV.** Representative hematoxylin-eosin-stained images of heart and liver sections on day 17 after immunization (*n* = 5 mice per group and ten pictures collected for each). 10× magnification.

**
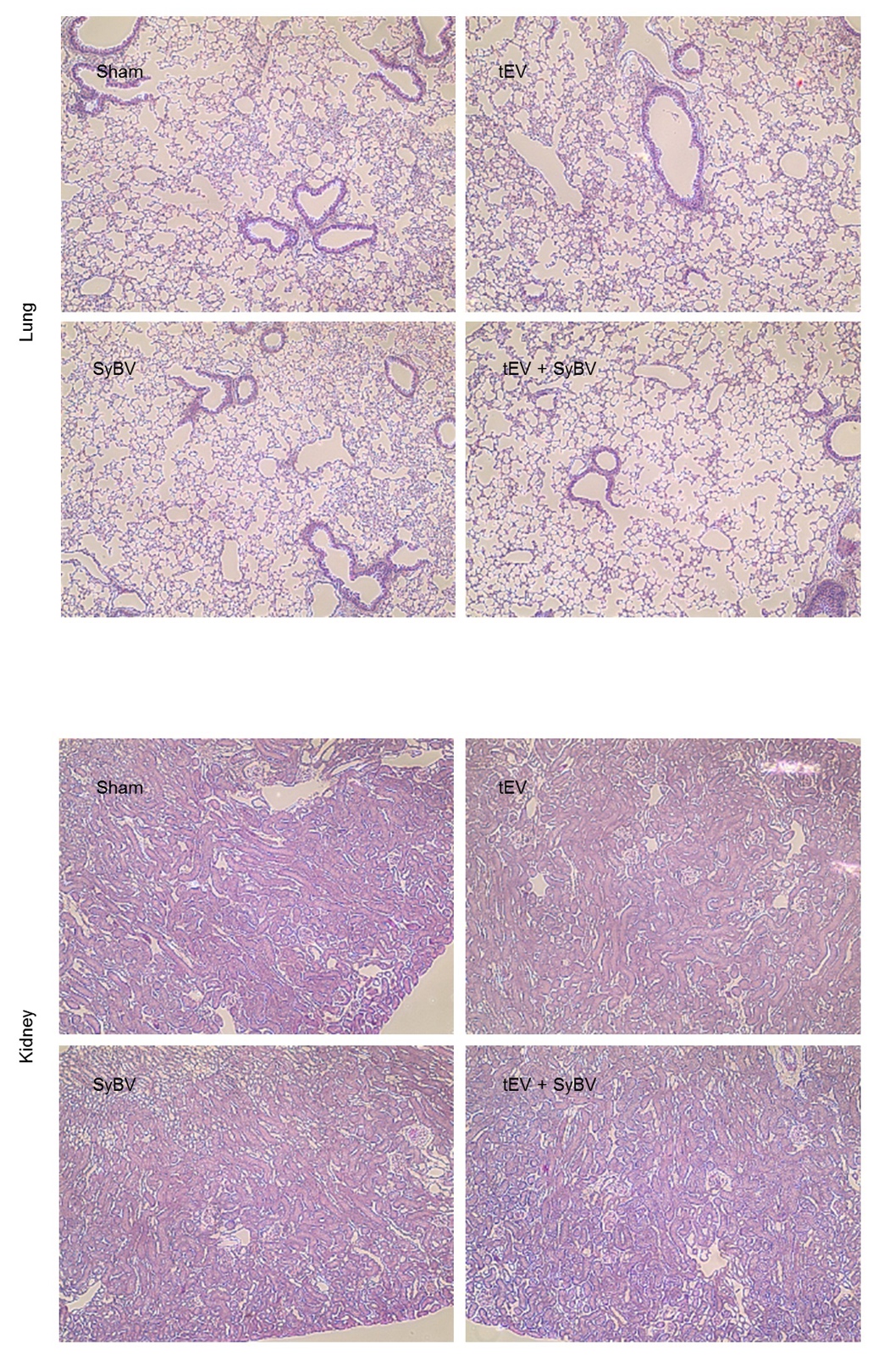
**

**Supplementary Figure 13. Safety profiles of mice immunized with tEV and SyBV.** Representative hematoxylin-eosin-stained images of lung and kidney sections on day 17 after immunization (*n* = 5 mice per group and ten pictures collected for each). 10× magnification.

**
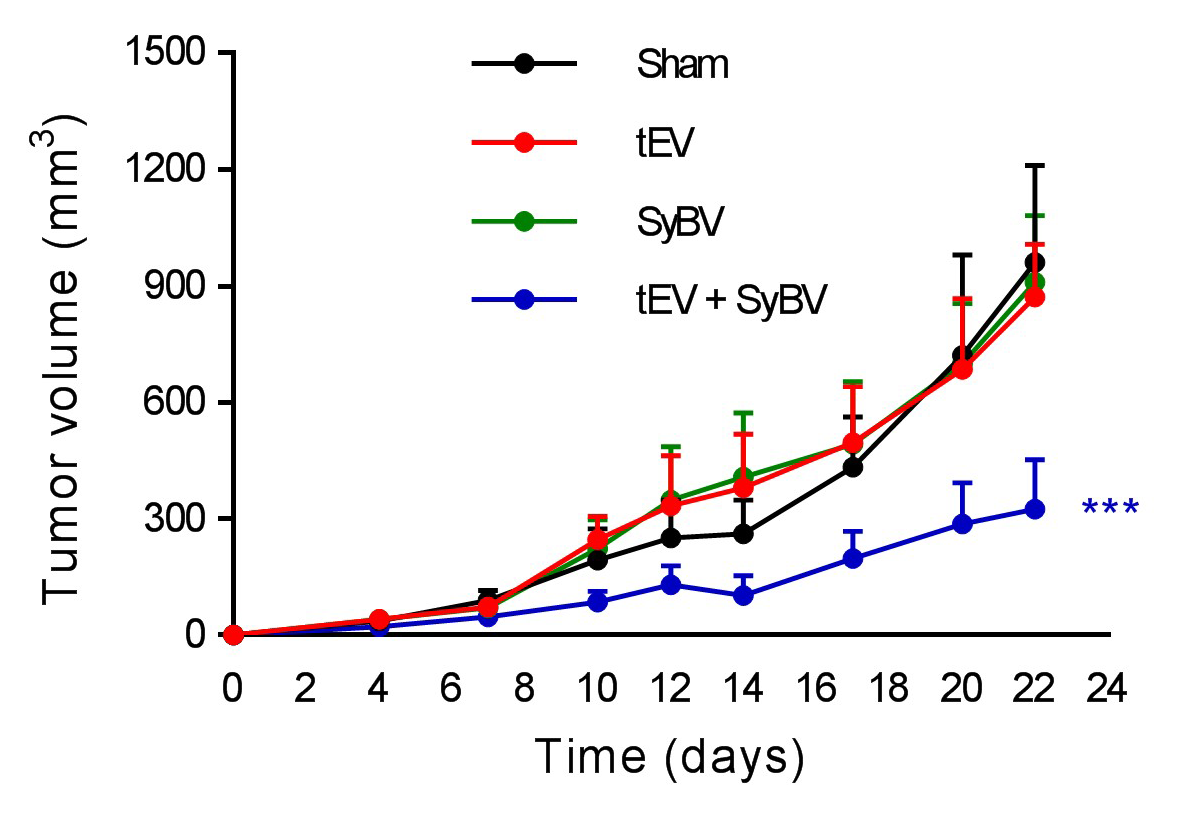
**

**Supplementary Figure 14. Therapeutic immunization with tEV and SyBV in CT26 tumor-bearing mice.** Mice were s.c. immunized with tEV (5 × 10^9^) and SyBV (5 × 10^9^) five times at 3-day intervals following CT26 inoculation (*n* = 5). Data are presented as mean ± s.e.m. ^***^*P* < 0.001; by one-way ANOVA with Tukey’s post test versus sham group.

**Supplementary Table 1. Proteins identified from *E. coli*-derived OMV and SyBV.**

| **Index** | **Protein name** | **UniProt accession** | **Gene symbol** |
| --- | --- | --- | --- |
| ***Exclusively identified proteins in OMV*** | | | |
| 1 | NADH dehydrogenase | P00393 | ndh |
| 2 | Isoleucine tRNA ligase | P00956 | ileS |
| 3 | Carbamoyl-phosphate synthase large chain | P00968 | carB |
| 4 | Penicillin-binding protein 1A | P02918 | mrcA |
| 5 | Penicillin-binding protein 1B | P02919 | mrcB |
| 6 | Ribose import binding protein RbsB | P02925 | rbsB |
| 7 | Maltose operon periplasmic protein | P03841 | malM |
| 8 | DNA topoisomerase 1 | P06612 | topA |
| 9 | Phenylalanine--tRNA ligase alpha subunit | P08312 | pheS |
| 10 | D-alanyl-D-alanine carboxypeptidase DacC | P08506 | dacC |
| 11 | UvrABC system protein A | P0A698 | uvrA |
| 12 | Glycerol kinase | P0A6F3 | glpK |
| 13 | 4-hydroxy-tetrahydrodipicolinate synthase | P0A6L2 | dapA |
| 14 | Glucose-6-phosphate isomerase | P0A6T1 | pgi |
| 15 | Glucose-1-phosphate adenylyltransferase | P0A6V1 | glgC |
| 16 | Translation initiation factor IF-3 | P0A707 | infC |
| 17 | 2-dehydro-3-deoxyphosphooctonate aldolase | P0A715 | kdsA |
| 18 | Ribose-phosphate pyrophosphokinase | P0A717 | prs |
| 19 | Glucosamine-6-phosphate deaminase | P0A759 | nagB |
| 20 | Transcription antitermination protein NusB | P0A780 | nusB |
| 21 | Aspartate carbamoyltransferase catalytic subunit | P0A786 | pyrB |
| 22 | Pyridoxine 5'-phosphate synthase | P0A794 | pdxJ |
| 23 | Inorganic pyrophosphatase | P0A7A9 | ppa |
| 24 | Adenylosuccinate synthetase | P0A7D4 | purA |
| 25 | 30S ribosome-binding factor | P0A7G2 | rbfA |
| 26 | Protein RecA | P0A7G6 | recA |
| 27 | 50S ribosomal protein L19 | P0A7K6 | rplS |
| 28 | 50S ribosomal protein L28 | P0A7M2 | rpmB |
| 29 | Ribose-5-phosphate isomerase A | P0A7Z0 | rpiA |
| 30 | Protein TolB | P0A855 | tolB |
| 31 | Serine--tRNA ligase | P0A8L1 | serS |
| 32 | Met repressor | P0A8U6 | metJ |
| 33 | Nucleoside-specific channel-forming protein Tsx | P0A927 | tsx |
| 34 | Bacterial non-heme ferritin | P0A998 | ftnA |
| 35 | Ferric uptake regulation protein | P0A9A9 | fur |
| 36 | Inducible lysine decarboxylase | P0A9H3 | cadA |
| 37 | Pyrroline-5-carboxylate reductase | P0A9L8 | proC |
| 38 | Acetyl-coenzyme A carboxylase carboxyl transferase subunit beta | P0A9Q5 | accD |
| 39 | Formate dehydrogenase-O iron-sulfur subunit | P0AAJ5 | fdoH |
| 40 | Probable L,D-transpeptidase YbiS | P0AAX8 | ybiS |
| 41 | Uncharacterized protein YcbK | P0AB06 | ycbK |
| 42 | Citrate synthase | P0ABH7 | gltA |
| 43 | Chaperone SurA | P0ABZ6 | surA |
| 44 | Glutaredoxin 2 | P0AC59 | grxB |
| 45 | Delta-aminolevulinic acid dehydratase | P0ACB2 | hemB |
| 46 | Hydrogenase-1 large chain | P0ACD8 | hyaB |
| 47 | Hydrogenase-2 large chain | P0ACE0 | hybC |
| 48 | DNA-binding protein StpA | P0ACG1 | stpA |
| 49 | Inosine-5'-monophosphate dehydrogenase | P0ADG7 | guaB |
| 50 | Uncharacterized protein YiaF | P0ADK0 | yiaF |
| 51 | Uncharacterized protein YggN | P0ADS9 | yggN |
| 52 | Protein YgiW | P0ADU5 | ygiW |
| 53 | Lipopolysaccharide export system protein LptA | P0ADV1 | lptA |
| 54 | 30S ribosomal protein S15 | P0ADZ4 | rpsO |
| 55 | Periplasmic protein CpxP | P0AE85 | cpxP |
| 56 | D-alanyl-D-alanine carboxypeptidase DacA | P0AEB2 | dacA |
| 57 | Thiol:disulfide interchange protein DsbA | P0AEG4 | dsbA |
| 58 | 3-oxoacyl-[acyl-carrier-protein] reductase FabG | P0AEK2 | fabG |
| 59 | L-cystine-binding protein FliY | P0AEM9 | fliY |
| 60 | UTP--glucose-1-phosphate uridylyltransferase | P0AEP3 | galU |
| 61 | Histidine-binding periplasmic protein | P0AEU0 | hisJ |
| 62 | Maltose-binding periplasmic protein | P0AEX9 | malE |
| 63 | NADH-quinone oxidoreductase subunit A | P0AFC3 | nuoA |
| 64 | NADH-quinone oxidoreductase subunit B | P0AFC7 | nuoB |
| 65 | Spermidine/putrescine-binding periplasmic protein | P0AFK9 | potD |
| 66 | GTP cyclohydrolase 1 type 2 homolog | P0AFP6 | ybgI |
| 67 | Negative modulator of initiation of replication | P0AFY8 | seqA |
| 68 | sn-glycerol-3-phosphate-binding periplasmic protein UgpB | P0AG80 | ugpB |
| 69 | Phosphate-binding protein PstS | P0AG82 | pstS |
| 70 | Protein translocase subunit SecF | P0AG93 | secF |
| 71 | Proline/betaine transporter | P0C0L7 | proP |
| 72 | Small-conductance mechanosensitive channel | P0C0S1 | mscS |
| 73 | Endo-type membrane-bound lytic murein transglycosylase A | P0C960 | emtA |
| 74 | Uridine phosphorylase | P12758 | udp |
| 75 | Xaa-Pro aminopeptidase | P15034 | pepP |
| 76 | Thiosulfate-binding protein | P16700 | cysP |
| 77 | Glutamine--fructose-6-phosphate aminotransferase | P17169 | glmS |
| 78 | UDP-N-acetylglucosamine--N-acetylmuramyl-(pentapeptide) pyrophosphoryl-undecaprenol N-acetylglucosamine transferase | P17443 | murG |
| 79 | NAD/NADP-dependent betaine aldehyde dehydrogenase | P17445 | betB |
| 80 | Glucose-1-phosphatase | P19926 | agp |
| 81 | Probable hydrolase YcaC | P21367 | ycaC |
| 82 | Aspartate--tRNA ligase | P21889 | aspS |
| 83 | UPF0701 protein YicC | P23839 | yicC |
| 84 | Periplasmic oligopeptide-binding protein | P23843 | oppA |
| 85 | Lactaldehyde dehydrogenase | P25553 | aldA |
| 86 | Transketolase 1 | P27302 | tktA |
| 87 | Cytoskeleton protein RodZ | P27434 | rodZ |
| 88 | Cell division protein FtsN | P29131 | ftsN |
| 89 | Flagellar hook-associated protein 3 | P29744 | flgL |
| 90 | Putative ABC transporter arginine-binding protein 2 | P30859 | artI |
| 91 | Uncharacterized HTH-type transcriptional regulator YafC | P30864 | yafC |
| 92 | Glycine dehydrogenase | P33195 | gcvP |
| 93 | L-lactate dehydrogenase | P33232 | lldD |
| 94 | Flagellar hook-associated protein 1 | P33235 | flgK |
| 95 | Periplasmic beta-glucosidase | P33363 | bglX |
| 96 | Periplasmic nitrate reductase | P33937 | napA |
| 97 | PTS system trehalose-specific EIIBC component | P36672 | treB |
| 98 | Phosphoglucomutase | P36938 | pgm |
| 99 | Cyclic di-GMP-binding protein | P37652 | bcsB |
| 100 | Ribosomal large subunit pseudouridine synthase B | P37765 | rluB |
| 101 | Periplasmic pH-dependent serine endoprotease DegQ | P39099 | degQ |
| 102 | Translocation and assembly module subunit TamB | P39321 | tamB |
| 103 | Isoaspartyl dipeptidase | P39377 | iadA |
| 104 | Cell division coordinator CpoB | P45955 | cpoB |
| 105 | Lipid A export ATP-binding/permease protein MsbA | P60752 | msbA |
| 106 | Elongation factor 4 | P60785 | lepA |
| 107 | N-acetylmuramoyl-L-alanine amidase AmiC | P63883 | amiC |
| 108 | Autonomous glycyl radical cofactor | P68066 | grcA |
| 109 | Maltose/maltodextrin import ATP-binding protein MalK | P68187 | malK |
| 110 | Sec-independent protein translocase protein TatB | P69425 | tatB |
| 111 | Glutamate decarboxylase beta | P69910 | gadB |
| 112 | Flagellar hook protein FlgE | P75937 | flgE |
| 113 | Uncharacterized protein YncE | P76116 | yncE |
| 114 | Autoinducer 2-binding protein LsrB | P76142 | lsrB |
| 115 | Uncharacterized protein YdiY | P76206 | ydiY |
| 116 | Lipid A 1-diphosphate synthase | P76445 | lpxT |
| 117 | Uncharacterized protein YfdP | P76512 | yfdP |
| 118 | UPF0070 protein YfgM | P76576 | yfgM |
| 119 | Bifunctional protein HldE | P76658 | hldE |
| 120 | Thiol:disulfide interchange protein DsbG | P77202 | dsbG |
| 121 | Gamma-aminobutyraldehyde dehydrogenase | P77674 | prr |
| 122 | Probable dimethyl sulfoxide reductase chain YnfF | P77783 | ynfF |
| 123 | L-glyceraldehyde 3-phosphate reductase | Q46851 | gpr |
| 124 | Uncharacterized protein YqiC | Q46868 | yqiC |
|  | | | |
| ***Exclusively identified proteins in SyBV*** | | | |
| 1 | PTS system mannitol-specific EIICBA component | P00550 | mtlA |
| 2 | Signal peptidase I | P00803 | lepB |
| 3 | Alanine--tRNA ligase | P00957 | alaS |
| 4 | Lipoprotein 28 | P04846 | nlpA |
| 5 | ATP-dependent 6-phosphofructokinase isozyme 2 | P06999 | pfkB |
| 6 | 1,4-alpha-glucan branching enzyme GlgB | P07762 | glgB |
| 7 | Glycerol-3-phosphate transporter | P08194 | glpT |
| 8 | Protease 4 | P08395 | sppA |
| 9 | PTS system N-acetylglucosamine-specific EIICBA component | P09323 | nagE |
| 10 | Acyl carrier protein ( | P0A6A8 | acpP |
| 11 | 10 kDa chaperonin | P0A6F9 | groS |
| 12 | D-amino acid dehydrogenase | P0A6J5 | dadA |
| 13 | Phosphopentomutase | P0A6K6 | deoB |
| 14 | 33 kDa chaperonin | P0A6Y5 | hslO |
| 15 | Divalent metal cation transporter MntH | P0A769 | mntH |
| 16 | 50S ribosomal protein L7/L12 (L8) | P0A7K2 | rplL |
| 17 | Triosephosphate isomerase | P0A858 | tpiA |
| 18 | Transaldolase A | P0A867 | talA |
| 19 | UPF0234 protein YajQ | P0A8E7 | yajQ |
| 20 | Regulator of ribonuclease activity A | P0A8R0 | rraA |
| 21 | Outer membrane protein W | P0A915 | ompW |
| 22 | Cold shock-like protein CspE | P0A972 | cspE |
| 23 | Cell division protein FtsZ | P0A9A6 | ftsZ |
| 24 | Uncharacterized ABC transporter ATP-binding protein YbhF | P0A9U1 | ybhF |
| 25 | Cold shock protein CspA | P0A9X9 | cspA |
| 26 | Transcriptional regulatory protein OmpR | P0AA16 | ompR |
| 27 | Protein QmcA | P0AA53 | qmcA |
| 28 | D-serine/D-alanine/glycine transporter | P0AAE0 | cycA |
| 29 | High-affinity choline transport protein | P0ABC9 | betT |
| 30 | Cytochrome bd-I ubiquinol oxidase subunit 2 | P0ABK2 | cydB |
| 31 | Coenzyme A biosynthesis bifunctional protein CoaBC | P0ABQ0 | coaB |
| 32 | Fumarate reductase iron-sulfur subunit | P0AC47 | frdB |
| 33 | Protoporphyrinogen IX dehydrogenase | P0ACB4 | hemG |
| 34 | cAMP-activated global transcriptional regulator CRP | P0ACJ8 | crp |
| 35 | Ribosome-associated inhibitor A | P0AD49 | raiA |
| 36 | Inhibitor of vertebrate lysozyme | P0AD59 | ivy |
| 37 | 50S ribosomal protein L23 | P0ADZ0 | rplW |
| 38 | Sec translocon accessory complex subunit YajC | P0ADZ7 | yajC |
| 39 | Serine endoprotease DegS | P0AEE3 | degS |
| 40 | 7-alpha-hydroxysteroid dehydrogenase | P0AET8 | hdhA |
| 41 | Lipoprotein NlpI | P0AFB1 | nlpI |
| 42 | Low-affinity inorganic phosphate transporter 1 | P0AFJ7 | pitA |
| 43 | Metalloprotease PmbA | P0AFK0 | pmbA |
| 44 | ECA polysaccharide chain length modulation protein | P0AG00 | wzzE |
| 45 | Probable protease SohB | P0AG14 | sohB |
| 46 | Single-stranded DNA-binding protein | P0AGE0 | ssb |
| 47 | Peroxiredoxin OsmC | P0C0L2 | osmC |
| 48 | Respiratory nitrate reductase 1 beta chain | P11349 | narH |
| 49 | Carbon starvation protein A | P15078 | cstA |
| 50 | Bifunctional purine biosynthesis protein PurH | P15639 | purH |
| 51 | Quinoprotein glucose dehydrogenase | P15877 | gcd |
| 52 | Proline--tRNA ligase | P16659 | proS |
| 53 | PTS system fructose-specific EIIB'BC component | P20966 | fruA |
| 54 | Xaa-Pro dipeptidase | P21165 | pepQ |
| 55 | Biosynthetic arginine decarboxylase | P21170 | speA |
| 56 | Ribonuclease R | P21499 | rnr |
| 57 | Apolipoprotein N-acyltransferase | P23930 | lnt |
| 58 | Probable aminoglycoside efflux pump | P24177 | acrD |
| 59 | Aconitate hydratase A | P25516 | acnA |
| 60 | Aminomethyltransferase | P27248 | gcvT |
| 61 | Bacteriophage adsorption protein A | P31600 | nfrA |
| 62 | Protein/nucleic acid deglycase 1 | P31658 | hchA |
| 63 | Uncharacterized lipoprotein YdeK | P32051 | ydeK |
| 64 | Uncharacterized Na(+)/H(+) exchanger YjcE | P32703 | yjcE |
| 65 | Glucans biosynthesis protein G | P33136 | mdoG |
| 66 | L-2-hydroxyglutarate oxidase LhgO | P37339 | lhgO |
| 67 | Glutamate/aspartate import solute-binding protein | P37902 | gltI |
| 68 | Lipoprotein BsmA | P39297 | bsmA |
| 69 | Inner membrane protein YjiY | P39396 | yjiY |
| 70 | Putative cation/proton antiporter YbaL | P39830 | ybaL |
| 71 | Lipoprotein NlpE | P40710 | nlpE |
| 72 | Heat shock protein HslJ | P52644 | hslJ |
| 73 | 2,3-bisphosphoglycerate-dependent phosphoglycerate mutase | P62707 | gpmA |
| 74 | UPF0114 protein YqhA | P67244 | yqhA |
| 75 | Uncharacterized lipoprotein YbjP | P75818 | ybjP |
| 76 | Macrolide export protein MacA | P75830 | macA |
| 77 | UPF0482 protein YnfB | P76170 | ynfB |
| 78 | Uncharacterized protein YdjY | P76220 | ydjY |
| 79 | Uncharacterized protein YebT | P76272 | yebT |
| 80 | Multidrug resistance protein MdtA | P76397 | mdtA |
| 81 | Multidrug resistance protein MdtB | P76398 | mdtB |
| 82 | UPF0339 protein YegP | P76402 | yegP |
| 83 | Uncharacterized protein YegR | P76406 | yegR |
| 84 | 2-methylcitrate dehydratase | P77243 | prpD |
| 85 | Probable iron export permease protein FetB | P77307 | fetB |
| 86 | Mechanosensitive channel MscK | P77338 | mscK |
| 87 | 2-methylisocitrate lyase | P77541 | prpB |
| 88 | Uncharacterized oxidoreductase YajO | P77735 | yajO |
| 89 | Acyl-coenzyme A dehydrogenase | Q47146 | fadE |
|  | | | |
| ***Common proteins*** | | | |
| 1 | Protease 7 | P09169 | ompT |
| 2 | NAD(P) transhydrogenase subunit beta | P0AB67 | pntB |
| 3 | Protein DcrB | P0AEE1 | dcrB |
| 4 | Uncharacterized protein YqjD | P64581 | yqjD |
| 5 | Uncharacterized protein YibT | Q2M7R5 | yibT |
| 6 | Flagellin | P04949 | fliC |
| 7 | Valine--tRNA ligase | P07118 | valS |
| 8 | Respiratory nitrate reductase 1 alpha chain | P09152 | narG |
| 9 | Formate acetyltransferase 1 | P09373 | pflB |
| 10 | Translation initiation factor IF-2 | P0A705 | infB |
| 11 | 50S ribosomal protein L10 | P0A7J3 | rplJ |
| 12 | 50S ribosomal protein L11 | P0A7J7 | rplK |
| 13 | 50S ribosomal protein L9 | P0A7R1 | rplI |
| 14 | 30S ribosomal protein S4 | P0A7V8 | rpsD |
| 15 | Lysine--tRNA ligase, heat inducible | P0A8N5 | lysU |
| 16 | MltA-interacting protein | P0A908 | mipA |
| 17 | ATP synthase gamma chain | P0ABA6 | atpG |
| 18 | DNA-binding protein HU-alpha | P0ACF0 | hupA |
| 19 | DNA-binding protein HU-beta | P0ACF4 | hupB |
| 20 | 50S ribosomal protein L14 | P0ADY3 | rplN |
| 21 | Septum site-determining protein MinD | P0AEZ3 | minD |
| 22 | 4-aminobutyrate aminotransferase GabT | P22256 | gabT |
| 23 | Uncharacterized protein YpfJ | P64429 | ypfJ |
| 24 | Cytosol aminopeptidase | P68767 | pepA |
| 25 | PTS system mannose-specific EIID component | P69805 | manZ |
| 26 | Uncharacterized protein YfeY | P76537 | yfeY |
| 27 | Uncharacterized lipoprotein YbaY | P77717 | ybaY |
| 28 | Outer membrane protein assembly factor BamB | P77774 | bamB |
| 29 | 30S ribosomal protein S6 | P02358 | rpsF |
| 30 | Pyruvate dehydrogenase | P07003 | poxB |
| 31 | Phosphoenolpyruvate-protein phosphotransferase | P08839 | ptsI |
| 32 | Elongation factor G | P0A6M8 | fusA |
| 33 | ATP-dependent 6-phosphofructokinase isozyme 1 | P0A796 | pfkA |
| 34 | 50S ribosomal protein L20 | P0A7L3 | rplT |
| 35 | Trigger factor | P0A850 | tig |
| 36 | NAD(P)H dehydrogenase | P0A8G6 | wrbA |
| 37 | Outer membrane protein assembly factor BamC | P0A903 | bamC |
| 38 | Phospholipase A1 | P0A921 | pldA |
| 39 | Membrane-bound lytic murein transglycosylase A | P0A935 | mltA |
| 40 | 2,3,4,5-tetrahydropyridine-2,6-dicarboxylate N-succinyltransferase | P0A9D8 | dapD |
| 41 | Isocitrate lyase | P0A9G6 | aceA |
| 42 | Energy-dependent translational throttle protein EttA | P0A9W3 | ettA |
| 43 | Rod shape-determining protein MreB | P0A9X4 | mreB |
| 44 | Fructose-bisphosphate aldolase class 2 | P0AB71 | fbaA |
| 45 | 2-amino-3-ketobutyrate coenzyme A ligase | P0AB77 | kbl |
| 46 | ATP synthase subunit b | P0ABA0 | atpF |
| 47 | Outer membrane protein assembly factor BamD | P0AC02 | bamD |
| 48 | Bifunctional protein GlmU | P0ACC7 | glmU |
| 49 | Uncharacterized lipoprotein YajG | P0ADA5 | yajG |
| 50 | Uncharacterized lipoprotein YifL | P0ADN6 | yifL |
| 51 | UPF0441 protein YgiB | P0ADT2 | ygiB |
| 52 | Inner membrane protein YhcB | P0ADW3 | yhcB |
| 53 | Enoyl-[acyl-carrier-protein] reductase | P0AEK4 | fabI |
| 54 | Glutamine-binding periplasmic protein | P0AEQ3 | glnH |
| 55 | 2-oxoglutarate dehydrogenase E1 component | P0AFG3 | sucA |
| 56 | Pyruvate dehydrogenase E1 component | P0AFG8 | aceE |
| 57 | Phage shock protein A | P0AFM6 | pspA |
| 58 | Uncharacterized oxidoreductase YghA | P0AG84 | yghA |
| 59 | Periplasmic serine endoprotease DegP | P0C0V0 | degP |
| 60 | Elongation factor Tu 2 | P0CE48 | tufB |
| 61 | Ribonuclease E | P21513 | rne |
| 62 | Periplasmic dipeptide transport protein | P23847 | dppA |
| 63 | Biotin carboxylase | P24182 | accC |
| 64 | Membrane protein insertase YidC | P25714 | yidC |
| 65 | Transketolase 2 | P33570 | tktB |
| 66 | Molybdate-binding protein ModA | P37329 | modA |
| 67 | 50S ribosomal protein L3 | P60438 | rplC |
| 68 | 50S ribosomal protein L24 | P60624 | rplX |
| 69 | Outer-membrane lipoprotein LolB | P61320 | lolB |
| 70 | Malate dehydrogenase | P61889 | mdh |
| 71 | Probable phospholipid ABC transporter-binding protein MlaD | P64604 | mlaD |
| 72 | Outer membrane lipoprotein RcsF | P69411 | rcsF |
| 73 | Sec-independent protein translocase protein TatA | P69428 | tatA |
| 74 | Maltodextrin phosphorylase | P00490 | malP |
| 75 | 50S ribosomal protein L15 | P02413 | rplO |
| 76 | Outer membrane protein TolC | P02930 | tolC |
| 77 | Outer membrane protein F | P02931 | ompF |
| 78 | Maltoporin | P02943 | lamB |
| 79 | Ferrienterobactin receptor | P05825 | fepA |
| 80 | Vitamin B12 transporter BtuB | P06129 | btuB |
| 81 | Ferrichrome outer membrane transporter/phage receptor | P06971 | fhuA |
| 82 | Outer membrane protein C | P06996 | ompC |
| 83 | NAD(P) transhydrogenase subunit alpha | P07001 | pntA |
| 84 | Isocitrate dehydrogenase | P08200 | icd |
| 85 | Elongation factor Ts | P0A6P1 | tsf |
| 86 | Chaperone protein DnaK | P0A6Y8 | dnaK |
| 87 | Chaperone protein HtpG | P0A6Z3 | htpG |
| 88 | Phosphoglycerate kinase | P0A799 | pgk |
| 89 | Transaldolase B | P0A870 | talB |
| 90 | Outer membrane lipoprotein Blc | P0A901 | blc |
| 91 | Outer membrane lipoprotein SlyB | P0A905 | slyB |
| 92 | Outer membrane protein A | P0A910 | ompA |
| 93 | Peptidoglycan-associated lipoprotein | P0A912 | pal |
| 94 | Outer membrane protein X | P0A917 | ompX |
| 95 | Outer membrane protein assembly factor BamA | P0A940 | bamA |
| 96 | Uncharacterized lipoprotein YeaY | P0AA91 | yeaY |
| 97 | Cytochrome bo(3) ubiquinol oxidase subunit 2 | P0ABJ1 | cyoA |
| 98 | Cytochrome bd-I ubiquinol oxidase subunit | P0ABJ9 | cydA |
| 99 | DNA protection during starvation protein | P0ABT2 | dps |
| 100 | Osmotically-inducible putative lipoprotein OsmE | P0ADB1 | osmE |
| 101 | LPS-assembly lipoprotein LptE | P0ADC1 | lptE |
| 102 | Translocation and assembly module subunit TamA | P0ADE4 | tamA |
| 103 | Uncharacterized protein YgaU | P0ADE6 | ygaU |
| 104 | Uncharacterized protein YidQ | P0ADM4 | yidQ |
| 105 | Multidrug efflux pump subunit AcrA | P0AE06 | acrA |
| 106 | Nucleoside permease NupC | P0AFF2 | nupC |
| 107 | 50S ribosomal protein L21 | P0AG48 | rplU |
| 108 | 30S ribosomal protein S1 | P0AG67 | rpsA |
| 109 | Long-chain fatty acid transport protein | P10384 | fadL |
| 110 | Fe(3+) dicitrate transport protein FecA | P13036 | fecA |
| 111 | Colicin I receptor | P17315 | cirA |
| 112 | Putative outer membrane porin protein NmpC | P21420 | nmpC |
| 113 | Phosphoenolpyruvate synthase | P23538 | ppsA |
| 114 | Multidrug export protein EmrA | P27303 | emrA |
| 115 | D-methionine-binding lipoprotein MetQ | P28635 | metQ |
| 116 | Uncharacterized lipoprotein YedD | P31063 | yedD |
| 117 | Multidrug efflux pump subunit AcrB | P31224 | acrB |
| 118 | LPS-assembly protein LptD | P31554 | lptD |
| 119 | Uncharacterized protein YddB | P31827 | yddB |
| 120 | Putative acyl-CoA dehydrogenase AidB | P33224 | aidB |
| 121 | Outer membrane protein slp | P37194 | slp |
| 122 | Multidrug resistance protein MdtE | P37636 | mdtE |
| 123 | Multidrug resistance protein MdtF | P37637 | mdtF |
| 124 | Cellulose synthase operon protein C | P37650 | bcsC |
| 125 | Probable lipoprotein YiaD | P37665 | yiaD |
| 126 | Antigen 43 | P39180 | flu |
| 127 | Paraquat-inducible protein B | P43671 | pqiB |
| 128 | 50S ribosomal protein L2 | P60422 | rplB |
| 129 | Probable glutamate/gamma-aminobutyrate antiporter | P63235 | gadC |
| 130 | Chaperone protein ClpB | P63284 | clpB |
| 131 | Uncharacterized lipoprotein YdcL | P64451 | ydcL |
| 132 | Uncharacterized lipoprotein YgdI | P65292 | ygdI |
| 133 | Major outer membrane prolipoprotein Lpp | P69776 | lpp |
| 134 | PTS system glucose-specific EIIA component | P69783 | crr |
| 135 | PTS system glucose-specific EIICB component | P69786 | ptsG |
| 136 | Aldehyde reductase YahK | P75691 | yahK |
| 137 | UPF0194 membrane protein YbhG | P75777 | ybhG |
| 138 | Catecholate siderophore receptor Fiu | P75780 | fiu |
| 139 | Probable TonB-dependent receptor YncD | P76115 | yncD |
| 140 | Protein YdgH | P76177 | ydgH |
| 141 | Probable phospholipid-binding lipoprotein MlaA | P76506 | mlaA |
| 142 | Copper-exporting P-type ATPase | Q59385 | copA |
| 143 | 6-phosphogluconate dehydrogenase | P00350 | gnd |
| 144 | Fumarate reductase flavoprotein subunit | P00363 | frdA |
| 145 | Aspartate aminotransferase | P00509 | aspC |
| 146 | RNA polymerase sigma factor RpoD | P00579 | rpoD |
| 147 | L-asparaginase 2 | P00805 | ansB |
| 148 | 30S ribosomal protein S7 | P02359 | rpsG |
| 149 | Polyribonucleotide nucleotidyltransferase | P05055 | pnp |
| 150 | Quinone-dependent D-lactate dehydrogenase | P06149 | dld |
| 151 | Dihydrolipoyllysine-residue acetyltransferase component of pyruvate dehydrogenase complex | P06959 | aceF |
| 152 | Putative uroporphyrinogen-III C-methyltransferase | P09127 | hemX |
| 153 | 60 kDa chaperonin | P0A6F5 | groL |
| 154 | ATP-dependent protease ATPase subunit HslU | P0A6H5 | hslU |
| 155 | Enolase | P0A6P9 | eno |
| 156 | Integration host factor subunit alpha | P0A6X7 | ihfA |
| 157 | 50S ribosomal protein L1 | P0A7L0 | rplA |
| 158 | 30S ribosomal protein S10 | P0A7R5 | rpsJ |
| 159 | 30S ribosomal protein S11 | P0A7R9 | rpsK |
| 160 | 30S ribosomal protein S13 | P0A7S9 | rpsM |
| 161 | 30S ribosomal protein S18 | P0A7T7 | rpsR |
| 162 | 30S ribosomal protein S20 | P0A7U7 | rpsT |
| 163 | 30S ribosomal protein S2 | P0A7V0 | rpsB |
| 164 | 30S ribosomal protein S5 | P0A7W1 | rpsE |
| 165 | 30S ribosomal protein S3 | P0A7V3 | rpsC |
| 166 | 30S ribosomal protein S8 | P0A7W7 | rpsH |
| 167 | 30S ribosomal protein S9 | P0A7X3 | rpsI |
| 168 | DNA-directed RNA polymerase subunit alpha | P0A7Z4 | rpoA |
| 169 | Serine hydroxymethyltransferase | P0A825 | glyA |
| 170 | Succinate--CoA ligase | P0A836 | sucC |
| 171 | Tryptophanase | P0A853 | tnaA |
| 172 | Probable transcriptional regulatory protein YebC | P0A8A0 | yebC |
| 173 | Uracil phosphoribosyltransferase | P0A8F0 | upp |
| 174 | DNA-directed RNA polymerase subunit beta' | P0A8T7 | rpoC |
| 175 | DNA-directed RNA polymerase subunit beta | P0A8V2 | rpoB |
| 176 | 3-oxoacyl-[acyl-carrier-protein] synthase 1 | P0A953 | fabB |
| 177 | Fructose-bisphosphate aldolase class 1 | P0A991 | fbaB |
| 178 | Glyceraldehyde-3-phosphate dehydrogenase A | P0A9B2 | gapA |
| 179 | Glutamine synthetase | P0A9C5 | glnA |
| 180 | Phosphate acetyltransferase | P0A9M8 | pta |
| 181 | Dihydrolipoyl dehydrogenase | P0A9P0 | lpdA |
| 182 | Aldehyde-alcohol dehydrogenase | P0A9Q7 | adhE |
| 183 | 50S ribosomal protein L13 | P0AA10 | rplM |
| 184 | ATP-dependent zinc metalloprotease FtsH | P0AAI3 | ftsH |
| 185 | Penicillin-binding protein activator LpoB | P0AB38 | lpoB |
| 186 | ATP synthase subunit alpha | P0ABB0 | atpA |
| 187 | ATP synthase subunit beta | P0ABB4 | atpD |
| 188 | Modulator of FtsH protease HflC | P0ABC3 | hflC |
| 189 | Modulator of FtsH protease HflK | P0ABC7 | hflK |
| 190 | Bacterioferritin | P0ABD3 | bfr |
| 191 | Cysteine synthase A | P0ABK5 | cysK |
| 192 | Purine nucleoside phosphorylase DeoD-type | P0ABP8 | deoD |
| 193 | Aspartate ammonia-lyase | P0AC38 | aspA |
| 194 | Succinate dehydrogenase flavoprotein subunit | P0AC41 | sdhA |
| 195 | Protein HemY | P0ACB7 | hemY |
| 196 | DNA-binding protein H-NS | P0ACF8 | hns |
| 197 | Pyruvate kinase I | P0AD61 | pykF |
| 198 | Murein hydrolase activator NlpD | P0ADA3 | nlpD |
| 199 | Uncharacterized protein YggE | P0ADS6 | yggE |
| 200 | Probable phospholipid-binding protein MlaC | P0ADV7 | mlaC |
| 201 | Peptidyl-prolyl cis-trans isomerase D | P0ADY1 | ppiD |
| 202 | 50S ribosomal protein L16 | P0ADY7 | rplP |
| 203 | D-galactose-binding periplasmic protein | P0AEE5 | mglB |
| 204 | Protein ElaB | P0AEH5 | elaB |
| 205 | Acid stress chaperone HdeB | P0AET2 | hdeB |
| 206 | Chaperone protein Skp | P0AEU7 | skp |
| 207 | Dihydrolipoyllysine-residue succinyltransferase component of 2-oxoglutarate dehydrogenase complex | P0AFG6 | sucB |
| 208 | Osmotically-inducible protein Y | P0AFH8 | osmY |
| 209 | Uncharacterized protein YibN | P0AG27 | yibN |
| 210 | 50S ribosomal protein L17 | P0AG44 | rplQ |
| 211 | 50S ribosomal protein L6 | P0AG55 | rplF |
| 212 | Protein translocase subunit SecD | P0AG90 | secD |
| 213 | Succinate--CoA ligase [ADP-forming] subunit alpha | P0AGE9 | sucD |
| 214 | 50S ribosomal protein L18 | P0C018 | rplR |
| 215 | Endolytic peptidoglycan transglycosylase RlpA | P10100 | rlpA |
| 216 | Cell division protein DamX | P11557 | damX |
| 217 | Catalase-peroxidase | P13029 | katG |
| 218 | Dimethyl sulfoxide reductase DmsA | P18775 | dmsA |
| 219 | Pyruvate kinase II | P21599 | pykA |
| 220 | NADH-quinone oxidoreductase subunit F | P31979 | nuoF |
| 221 | NADH-quinone oxidoreductase subunit C/D | P33599 | nuoC |
| 222 | Aconitate hydratase B | P36683 | acnB |
| 223 | Peptidase B | P37095 | pepB |
| 224 | Zinc/cadmium/lead-transporting P-type ATPase | P37617 | zntA |
| 225 | Uncharacterized protein YhiI | P37626 | yhiI |
| 226 | Penicillin-binding protein activator LpoA | P45464 | lpoA |
| 227 | FKBP-type peptidyl-prolyl cis-trans isomerase FkpA | P45523 | fkpA |
| 228 | Putative acyl-CoA thioester hydrolase YbhC | P46130 | ybhC |
| 229 | 50S ribosomal protein L4 | P60723 | rplD |
| 230 | 50S ribosomal protein L22 | P61175 | rplV |
| 231 | 50S ribosomal protein L5 | P62399 | rplE |
| 232 | UPF0325 protein YaeH | P62768 | yaeH |
| 233 | Uncharacterized protein YraP | P64596 | yraP |
| 234 | 30S ribosomal protein S21 | P68679 | rpsU |
| 235 | Outer membrane protein YfaZ | P76471 | yfaZ |
| 236 | Uncharacterized protein YfdQ | P76513 | yfdQ |
| 237 | NADP-dependent malic enzyme | P76558 | maeB |
| 238 | Protein YdgA | P77804 | ydgA |
| 239 | Uncharacterized lipoprotein YgeR | Q46798 | ygeR |
